# Role of S protein transmembrane domain mutations in the development of occult hepatitis B virus infection

**DOI:** 10.1080/22221751.2022.2114849

**Published:** 2022-09-21

**Authors:** Xinyi Jiang, Le Chang, Ying Yan, Huimin Ji, Huizhen Sun, Yingzi Xiao, Shi Song, Kaihao Feng, Abudulimutailipu Nuermaimaiti, Lunan Wang

**Affiliations:** a National Center for Clinical Laboratories, Institute of Geriatric Medicine, Chinese Academy of Medical Sciences, Beijing Hospital/National Center of Gerontology, Beijing, People’s Republic of China; b Beijing Engineering Research Center of Laboratory Medicine, Beijing Hospital, Beijing, People’s Republic of China; c Graduate School, Peking Union Medical College, Chinese Academy of Medical Sciences, People’s Republic of China

**Keywords:** Occult hepatitis B virus infection, transmembrane domain mutations, in vivo analysis, HBsAg secretion impairment, protein structure prediction

## Abstract

Occult HBV infection (OBI) is a special infection status during Hepatitis B virus (HBV) infection. The underlying mechanism of its occurrence remains unclear. This study conducted sequencing analysis on 104 OBI plasma samples and 524 HBsAg positive samples from 29 blood centres, and searched for high-frequency mutations in transmembrane domain (TMD) of S protein in the OBI population. Plasmids with TMD high-frequency mutations were constructed, *in vivo* and *in vitro* functional experiments were performed to investigate possible molecular mechanisms of OBI occurrence. We found 22 high-frequency TMD mutations in genotype B OBI strains. Among them, five mutations can lead to impairment of HBsAg secretion; seven mutations had accumulated intracellular HBsAg while extracellular HBsAg didn’t decrease compared to wildtype. This study chose C85R from TMD2, F220C, and F220Y from TMD4 for further exploration. Protein structure predication showed these three mutant HBsAg displayed changed hydrophilic properties and tended to accumulate in the phospholipid bilayer of cell membrane. Mutant HBsAg’s secretion disorder may induce OBI. On the other hand, V168A + V177A from TMD3 expressed increased HBsAg both in intracellular and extracellular levels. This mutation had most unstable natural conformation and may be inclined to transition into V177A or V168A + S174N + V177A. These three mutations were more prone to mixed infection, presenting a state of coexistence, thus approaching the impaired secretion pattern of OBI. This study demonstrated TMD mutations could contribute to the occurrence of OBI and provided a theoretical basis for OBI study and the functional cure of chronic hepatitis B virus infection.

## GRAPHICAL ABSTRACT

**Figure:**
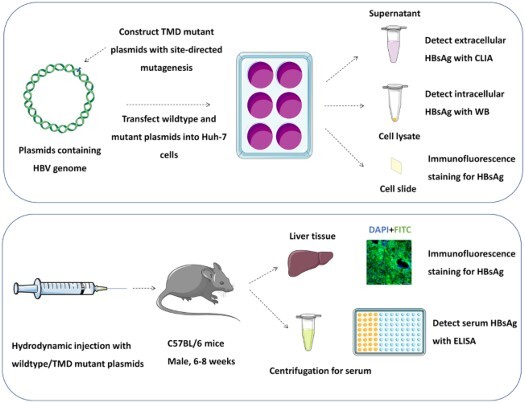

## Introduction

Hepatitis B virus (HBV) can establish persistent infections in humans. Due to the peculiar life cycle of HBV, a special infectious status is gaining attention. Occult HBV infection (OBI) is termed as hepatitis B surface antigen (HBsAg) negative people (blood tested by currently available assays) with replication-competent HBV DNA (cccDNA) in the liver and/or HBV DNA in the blood [1].

The prevalence of OBI was reported in a specific group. A meta-analysis found OBI prevalence in Chinese blood donors was 0.094% [2]. OBI detection rates ranged from 0.63% to 88.4% in HBV and human immunodeficiency virus (HIV) co-infected patients [3]. The prevalence of OBI was even higher in patients with progressive liver diseases, the rates were 40% to 75% in those with HCC [1]. Meanwhile, OBI is transmissible via blood transfusion, liver transplantation as well as mother-to-child routes [4–6]. It can reactivate HBV infection when the host immune response is compromised, as under chemotherapy and during organ transplantation. It may potentially contribute to progressive liver diseases and is considered an important risk factor for HCC [7–9]. Therefore, the endemicity and risks of OBI cannot be underestimated.

To get a better understanding of OBI, we need to investigate the mechanisms for its occurrence. Virus-host interactions play an important role in OBI occurrence and viral factors are hot spots for mainstream research. Many articles focused on mutations within major hydrophilic region (MHR) of HBsAg (residues 99–160) as MHR contains the α determinant, a core part for anti-HBs neutralization. A variety of mutations associated with OBI have been discovered [10–12]. Among them, G145R is the most well-known immune escape mutant, showing deficiency in HBsAg secretion, antigenicity and anti-HBs binding [13,14].

Nowadays, studies have extended sequence analyses outside the MHR region of the S gene [15]. HBsAg has four transmembrane domains (TMDs), which pack to form channels through the viral envelope and are involved in cell entry [16,17]. HBsAg is unable to anchor stably to the viral envelope and release freely from infected cells without TMDs. We previously found the significance of TMDs by replacing the original sites with the hydrophilic amino acid (aa) arginine (R). We discovered C85R, L87R, L88R, and C90R in TMD2 significantly impaired both HBsAg production and secretion [18]. In this study, we focused on the high-frequency mutations in the TMD of S protein from OBI blood donors, performed a comprehensive functional analysis of the selected TMD mutants *in vitro* and *in vivo* to investigate the possible mechanisms of OBI occurrence among Chinese voluntary blood donors.

## Materials and methods

### Enrolled patients and study sequences

During January 2010 to December 2013, 1261 blood samples characterized as HBsAg-/HBV DNA+ were transported to National Center for Clinical Laboratories in Beijing from 29 blood centres. Serological indicators including HBsAg, anti-HBs, HBeAg, anti-HBe, and anti-HBc were retested with ARCHITECT i2000 (Abbott Laboratories, USA) and HBV DNA was quantified with Cobas AmpliPrep TaqMan 2.0 (Roche, Switzerland). After excluding anti-HBc-/anti-HBs- samples, 918 HBsAg-/HBV DNA+ (viral load <200 IU/mL) samples were considered as OBI carriers. Among them, 250 OBI samples were randomly selected and S sequences were obtained from 104 samples. Meanwhile, HBsAg positive (HBsAg+) samples from these blood centers were enrolled and sequenced as control group [18]. 168 sequences of genotype C and 356 sequences of genotype B were included in HBsAg+ group. 51 and 53 blood donors infected with genotype C and genotype B OBI strains were confirmed respectively. Their S gene amplification products were connected to T vector, and monoclones were selected and sequenced. 220 monoclonal sequences of genotype C and 228 monoclonal sequences of genotype B were obtained.

### Selection and construction of candidate TMD mutations

Specific regions of four TMDs were showing below [19]: TMD1 (4–24 aa), TMD2 (80–98 aa), TMD3 (160–193 aa) and TMD4 (202–222 aa). Software Geneious Prime, Mega 7.0, and online software VESPA were used to analyse mutations of each TMD in the OBI group and HBsAg+ group. Point and combined mutations’ occurrence frequency were calculated. Mutational analysis was applied at both nucleotide and amino acids levels to ensure all the selected mutants come from real samples.

The 1.2×HBV construct (pBB4.5-HBV1.2, genotype C2, covered the HBV genome nucleotide 1389–3215 and 1–1969) and 1.3×HBV construct (pGEM-HBV1.3B, genotype B2, covered the HBV genome nucleotide 1013–3182 and 1–1986) was used to construct mutations. The plasmids were kindly provided by Professor Fengmin Lu from Peking University Health Science Center in China [20]. Site-directed mutagenesis was performed to generate different mutant HBsAg constructs using QuikChange Lightning Site-Directed Mutagenesis Kit (Agilent Technology, USA) according to the manufacturer’s instructions. The primers can refer to supplementary Tables 1–3. The constructs were subsequently confirmed by Sanger sequencing.

### Functional analysis and structure prediction

Detailed information is described in the Supporting Materials.

### Statistical analysis

SPSS 21.0 software and GraphPad Prism 8.0 were utilized for statistical analysis. *p* < 0.05 was considered statistically significant. Detailed statistical analyses are described in the Supporting Materials.

## Results

### Screening for high-frequency mutations

**Figure 1.**
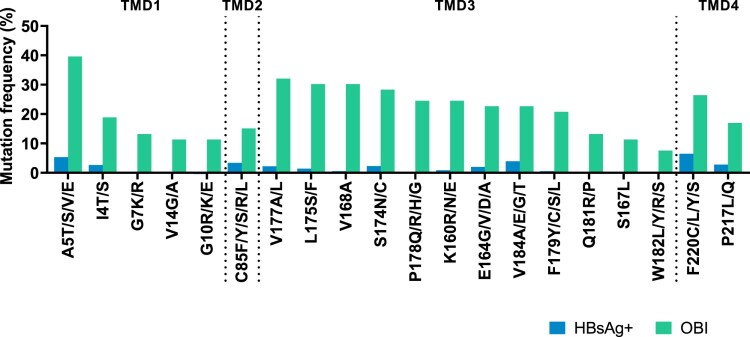
High-frequency mutations in four TMDs of genotype B OBI sequences. Mutations between OBI and HBsAg+ control groups were examined using Fisher's exact test (two-sided). Mutations were statistically appeared with high-frequency in the OBI group including 5 sites in TMD1 (the 4th, 5th, 7th, 10^th^, and 14th sites), 1 site in TMD2 (the 85th site), 12 sites in TMD3 (the 160th, 164th, 167th, 168th, 174th, 175th, 177th, 178th, 179th, 181th, 182th, and 184th sites), and 2 sites in TMD4 (the 217th and 220th sites). Each site may be substituted with various amino acids. The positions of four TMDs: TMD1 (4–24 aa), TMD2 (80–98 aa), TMD3 (160–193 aa), and TMD4 (202–222 aa).

We found high-frequency OBI-related mutations in four TMDs (Supplementary figures 1 and 2). In genotype B (Figure 1), 20 aa positions in the OBI group showed significantly higher mutation rates than the HBsAg+ group (*p* < 0.05). In genotype C, there were nine high-frequency mutation sites (Supplementary figure 3). Different mutations may occur at the same site, we selected substitution with highest frequency at specific site to construct candidate mutations. Meanwhile, five pairs of mutations at the same site (A5E and A5T, E164G and E164A, V184A and V184E, W182L and W182R, F220C and F220Y) were chosen from genotype B to study whether the replacement of aa with different properties can affect HBsAg expression.

Combined aa substitutions may exert a greater effect on the structure and function of HBsAg, therefore, this study further selected combined mutations in the OBI group (Table 1). We discovered more high-frequency mutations in TMD3 were related to combined mutations. Among these mutations, L175S had a positive relationship with V168A, V177A, and V184A (Phi > 0.3, *p* < 0.001), V177A had a positive relationship with K160R, V168A, S174N, and L175S (Phi > 0.3, *p* < 0.001). According to the frequency of combined mutations, we selected 6 double mutations and 5 triple mutations for subsequent research.

**Table 1.** Selection of combined TMD mutations (Genotype B).

	Mutations	Occurrence in OBI donors (n)	Frequency of mutations (total = 53)	Involved TMD regions	Relationship between mutations	
					Phi	*p*
Double mutations	**L175S + V177A**	**11**	**20**.**8%**	**TMD3**	**0**.**313**	***p* < 0.001**
	**L175S + V168A**	**10**	**18**.**9%**	**TMD3**	**0**.**367**	***p* < 0.001**
	**V168A + V177A**	**9**	**17**.**0%**	**TMD3**	**0**.**367**	***p* < 0.001**
	**S174N + V177A**	**9**	**17**.**0%**	**TMD3**	**0**.**384**	***p* < 0.001**
	A5T + I4T	7	13.2%	TMD1	–	–
	A5T + L175S	7	13.2%	TMD1 + TMD3	–	–
	**P178Q + S174N**	**7**	**13**.**2%**	**TMD3**	**0**.**357**	***p* < 0.001**
	P178Q + V177A	7	13.2%	TMD3	−0.119	
	S174N + V168A	7	13.2%	TMD3	0.252	*p* < 0.001
	L175S + S174N	6	11.3%	TMD3	0.001	
	I4T + V168A	6	11.3%	TMD1 + TMD3	–	–
	V177A + V184A	6	11.3%	TMD3	0.243	*p* < 0.001
	**L175S + V184A**	**6**	**11**.**3%**	**TMD3**	**0**.**316**	***p* < 0.001**
Triple mutations	**L175S + V168A + V177A**	**7**	**13**.**2%**	**TMD3**		
	**P178Q + S174N + V177A**	**6**	**11**.**3%**	**TMD3**		
	**S174N + V168A + V177A**	**6**	**11**.**3%**	**TMD3**		
	**L175S + V177A + V184A**	**5**	**9**.**4%**	**TMD3**		
	**L175S + S174N + V177A**	**5**	**9**.**4%**	**TMD3**		

*Coefficient of binomial correlation (Phi) was used to evaluate the correlation between mutations. Co-variations with 0.3 < Phi <1 and *p* < 0.05 considered to be significant positive correlated mutations. The bold mutations were the combined TMD mutations we chose to construct.

### *In vitro* functional analysis of TMD mutations

**Figure 2.**
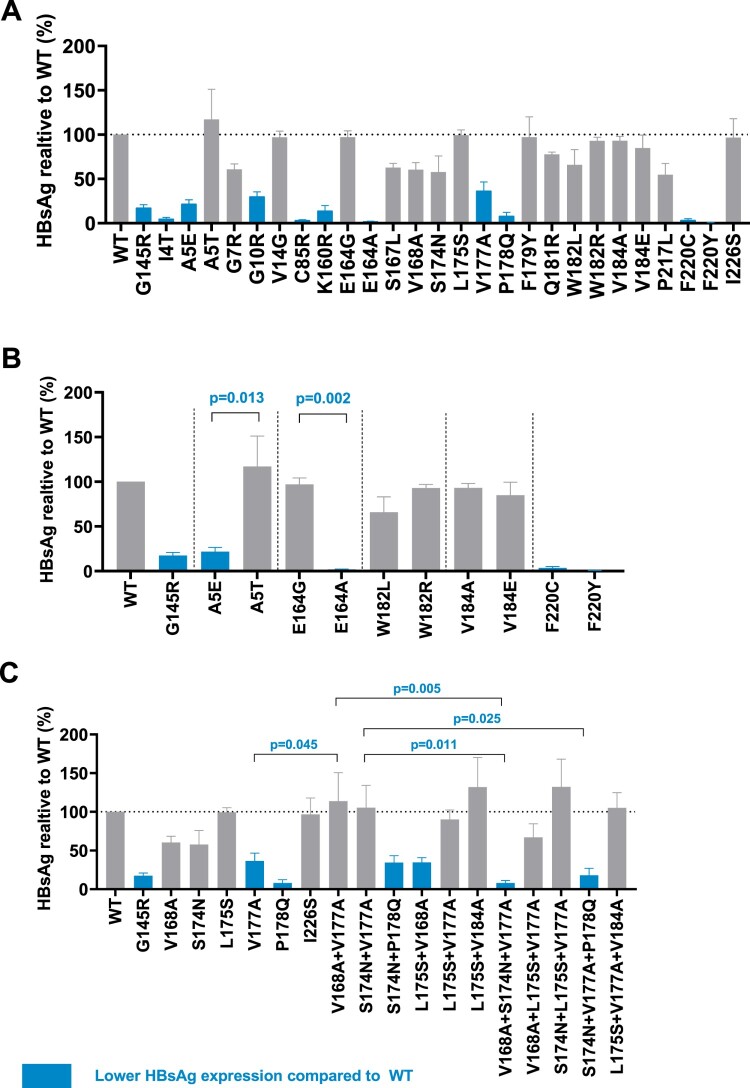
High-frequency TMD mutations of genotype B affect extracellular HBsAg expression. Extracellular HBsAg expression of wildtype or TMD mutants was detected by CLIA and normalized according to SEAP activity. Data were demonstrated as mean (SD) from three independent experiments. **(A)** Extracellular HBsAg expression of single point TMD mutation. **(B)** Extracellular HBsAg expression of different amino acid substitutions at the same site. **(C)** Extracellular HBsAg expression of combined TMD3 mutations and single point mutation involved in these combined mutations. Blue columns represented that mutant HBsAg expression was significantly lower than that of wildtype (examined by Kruskal–Wallis analysis). G145R is the positive control and I226S is the negative control.

This study transfected TMD mutant and wildtype HBV plasmids into Huh-7 cells, and detected extracellular and intracellular HBsAg expression. In genotype B, Figure 2(A) showed that several mutations’ (including mutations I4T, A5E, and G10R from TMD1; C85R from TMD2; K160R, V177A, and P178Q from TMD3; and F220Y and F220C from TMD4) extracellular HBsAg expression was significantly lower than that in wildtype. Figure 2(B) displayed different substitutions within the same site. Among them, A5T and E164G’s HBsAg expression did not decrease compared to wildtype, while A5E and E164A had lower extracellular HBsAg expression compared to wildtype and mutations at the same site. Lower HBsAg occurred when there were substitutions between amino A (alanine) and E (glutamic acid). At sites 182 and 184, the substitution of two amino acids did not significantly affect the expression of extracellular HBsAg. At the 220th site, both substitutions F220Y and F220C showed extremely low expression of HBsAg. In Figure 2(C), several combined mutations had a lower HBsAg expression, including mutations V168A + L175S, S174N + P178Q, V168A + S174N + V177A, and S174N + V177A + P178Q.

**Figure 3.**
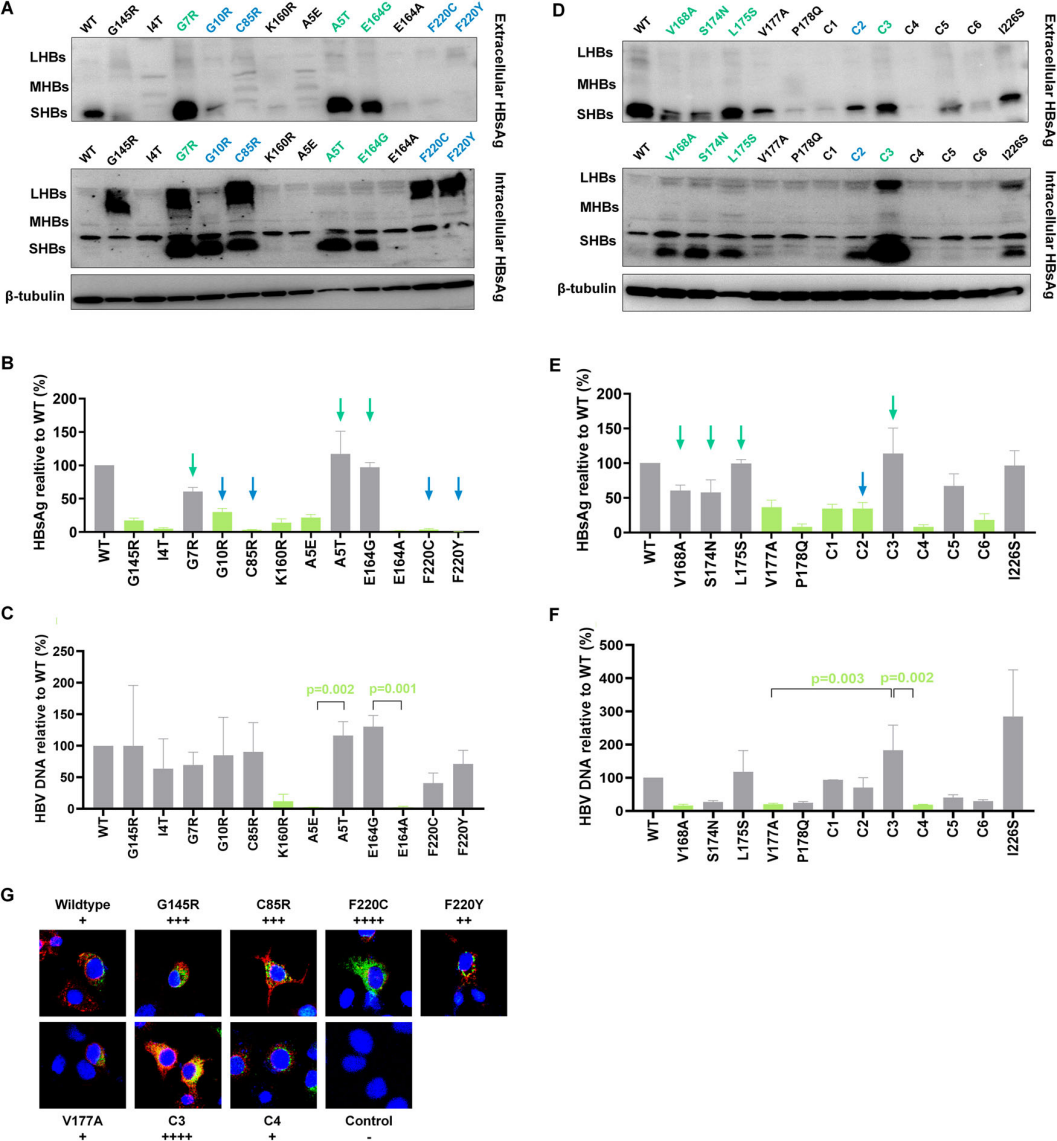
High-frequency TMD mutations of genotype B affect HBsAg secretion. 22 mutations were chosen for further study. The selection principles were below: (1) For single point mutation: the expression of extracellular HBsAg was significantly lower than that of wildtype; (2) For mutations at the same site: two mutations expressed significantly different level of HBsAg; (3) For combined mutations, we chose mutations expressed significantly different level of HBsAg than that of wildtype, and single mutations involved in these combined mutations. **(A)** 11 mutations from TMD1, TMD2 and TMD4 as well as wildtype, positive control G145R, their extracellular and intracellular HBsAg expression was detected by WB analysis. **(B)** CLIA detected extracellular HBsAg and **(C)** qRT-PCR detected extracellular HBV DNA in 11 mutations from **(A)**. Light green columns represented that mutant HBsAg or HBV DNA expression was significantly lower than that of wildtype (Kruskal–Wallis analysis). **(D)** 11 mutations from TMD3 as well as wildtype, negative control I226S, their extracellular and intracellular HBsAg expression was detected by WB. **(E)** CLIA detected extracellular HBsAg and **(F)** qRT-PCR detected extracellular HBV DNA in 11 mutations from **(D)**. The mutation names with blue colour/indicated with blue arrows represented that they exhibited an impaired HBsAg secretion with accumulated intracellular HBsAg and lower extracellular HBsAg. The mutations with green colour/indicated with green arrows represented that they had accumulated intracellular HBsAg, but the extracellular HBsAg didn’t decrease, and in some mutations even increase (V168A + V177A). Combination 1 (C1) represents V168A + L175S, Combination 2 (C2) represents S174N + P178Q, Combination 3 (C3) represents V168A + V177A, Combination 4 (C4) represents V168A + S174N + V177A, Combination 5 (C5) represents V168A + L175S + V177A, Combination 6 (C6) represents S174N + V177A + P178Q. **(G)** Immunofluorescence staining of HBsAg in Huh-7 cells transfected with wildtype and TMD mutant plasmids. Intracellular HBsAg was stained by FITC (green), and endoplasmic reticulum marker calnexin was stained by Alexa Fluor 647 (red). The cell nuclei were stained with DAPI (blue). Magnification, ×400. The original figures can refer to supplementary figure 8.

Based on the above results, we focused on 22 mutations in Figure 3. Among them, five mutations (G10R, C85R, F220C, F220Y, and S174N + P178Q) showed lower extracellular HBsAg and higher intracellular HBsAg compared to wildtype. Seven mutations’ (A5T, G7R, E164G, V168A, S174N, L175S, and V168A + V177A) extracellular HBsAg didn’t decrease while they exhibited higher intracellular HBsAg. We focused on C85R from TMD2, F220C, and F220Y from TMD4, which exhibited a relative higher level of intracellular HBsAg and their extracellular HBsAg was even undetected (Figure 3(A,B)). Figure 3(D,E) showed intracellular and extracellular HBsAg expression in TMD3 mutations. V168A + V177A (Combination 3) expressed large amount of HBsAg both in intracellular and extracellular levels. Add a mutation (forming V168A + S174N + V177A, Combination 4) or remove a mutation (forming V177A) based on V168A + V177A, both extracellular and intracellular HBsAg became lower. Moreover, the extracellular HBV DNA level was also higher in V168A + V177A compared to V177A and V168A + S174N + V177A (Figure 3F).

In genotype C, nine mutations showed lower extracellular HBsAg expression both in chemiluminescent immunoassay (CLIA) and western blot (WB) detection (Supplementary figure 4), while their intracellular HBsAg had a comparable level to wildtype. Moreover, the HBV DNA level was also lower in mutants compared to wildtype (Supplementary figure 5). The specific cause of decreased extracellular HBsAg is not clear, reduced viral protein synthesis, secretion impairment and protein instability can be the reasons. The mechanisms between genotype C TMD mutations and OBI were more complicated while some genotype B TMD mutations had remarkable intracellular HBsAg aggregation, their mechanisms leading to OBI is more explicit. As a result, we focused on genotype B TMD mutations in this research.

### *In vivo* functional analysis of TMD mutations

**Figure 4.**
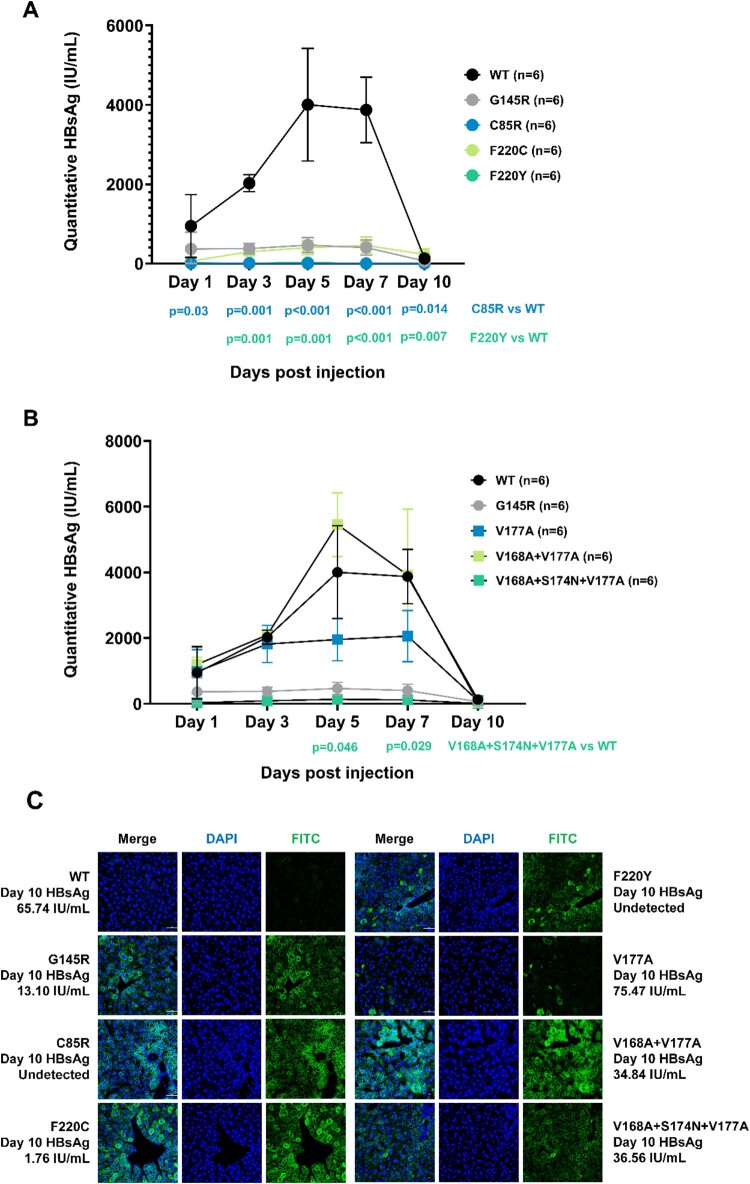
*In vivo* analysis for HBsAg detection. Six C57BL/6 mice were included in each group to construct HBV infection model by hydrodynamic injection with wildtype or TMD mutant plasmids. **(A and B)** Blood samples were collected from tail vein at Days 1, 3, 5, 7, 10 after injection. HBsAg from serum was detected by ELISA. The standard curve was drawn based on the results of the standard substance, and the quantification of HBsAg (IU/mL) was calculated. Kruskal-Wallis analysis was performed to compare the serum HBsAg level in each group. **(C)** Mice in each group were sacrificed on day 10 after injection for liver tissue. Frozen sections were stained with immunofluorescence assay, HBsAg was stained with FITC (green), and nuclei were stained with DAPI (blue). Confocal microscopy was used to capture the pictures (magnification ×400). On day 10, the serum HBsAg in the mice from specific group was labeled next to the pictures.

Specific mutations in genotype B were chosen for further studies to reveal their relationship with OBI. C85R, F220C, and F220Y showed impaired secretion, combined mutation V168A + V177A showed higher HBsAg expression both in extracellular and intracellular levels, compared to V168A + S174N + V177, V177A, and wildtype. The accumulation of HBsAg in Huh-7 cells was also observed in mutation C85R, F220C, F220Y and V168A + V177A in Figure 3(G). These six mutations were selected for *in vivo* analysis. We constructed HBV infection model in C57BL/6 mice (Figure 4). Serum HBsAg of mice was at a high level within 10 days in the wildtype group. On day 5 after injection, serum HBsAg reached the peak (about 4000 IU/mL). On day 7, serum HBsAg decreased slightly. While on day 10, serum HBsAg dropped to low level. In V168A + V177A group, serum HBsAg was also at a high level, and the average HBsAg expression exceeded the wildtype group on day 5. The expression of HBsAg in the V177A group was at an intermediate level within 10 days. While in the mutant groups, including the positive control G145R, C85R from TMD2, F220C and F220Y from TMD4, serum HBsAg was at a low level during the experiment. Especially, the expression of HBsAg in the C85R group was very low or undetected at all five time points, which was significantly lower than that in the wildtype group.

Immunofluorescence staining of the frozen section from mouse liver tissue was consistent with *in vitro* analysis. On day 10, serum HBsAg was over 60 IU/mL, while weak green fluorescence was detected in liver tissue from wildtype mouse. On day 10, HBsAg was undetected in serum, but the mouse from the C85R group and F220Y group showed clear and strong green fluorescence in liver tissue which indicated HBsAg’s aggregation. The mouse from the F220Y group also had a low-level HBsAg in serum (1.76 IU/mL) while intracellular HBsAg aggregation occurred. The mutation V168A + V177A from TMD3 expressed about 35 IU/mL serum HBsAg and had HBsAg aggregation in liver tissue. Its intracellular HBsAg expression was higher than that of the related V177A or V168A + S174N + V177A mutations, which was consistent with the *in vitro* experiments.

### Underlying mechanism of TMD mutations and impaired secretion

**Figure 5.**
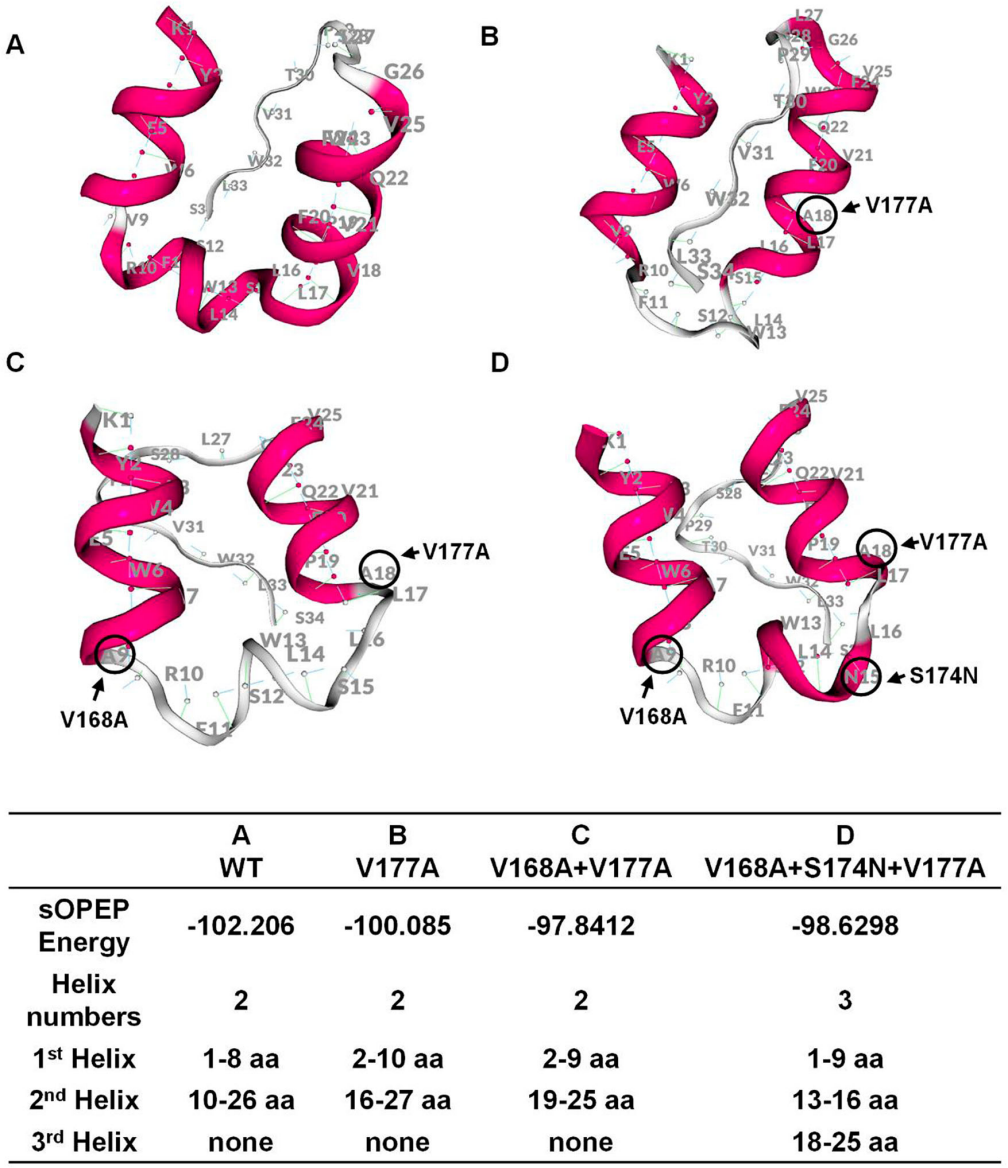
Structure prediction for transmembrane domain 3 (TMD3) of S protein. (A–D) PEP-FOLD was used to predict the protein structure of wildtype and mutant TMD3. The amino acids circled in black are the TMD3 mutations. In the table, the value of SOPEP Energy is negative. The larger this value is, the more energy is required to maintain the stability of the protein. The table also displays the helix number and the amino acid composition of each helix.

Pep-fold software was used to predict the protein structure of TMD3. In Figure 5, the conformation of TMD3 will change greatly compared to wildtype when more mutations are introduced. Lowest absolute sOPEP energy value of V168A + V177A revealed its natural conformation was most unstable, and we speculated that V168A + V177A may be inclined to remove one mutation (forming V177A) or increase one mutation (forming V168A + S174N + V177A) during the development of OBI, thus exhibiting a more typical secretion impaired pattern.

**Figure 6.**
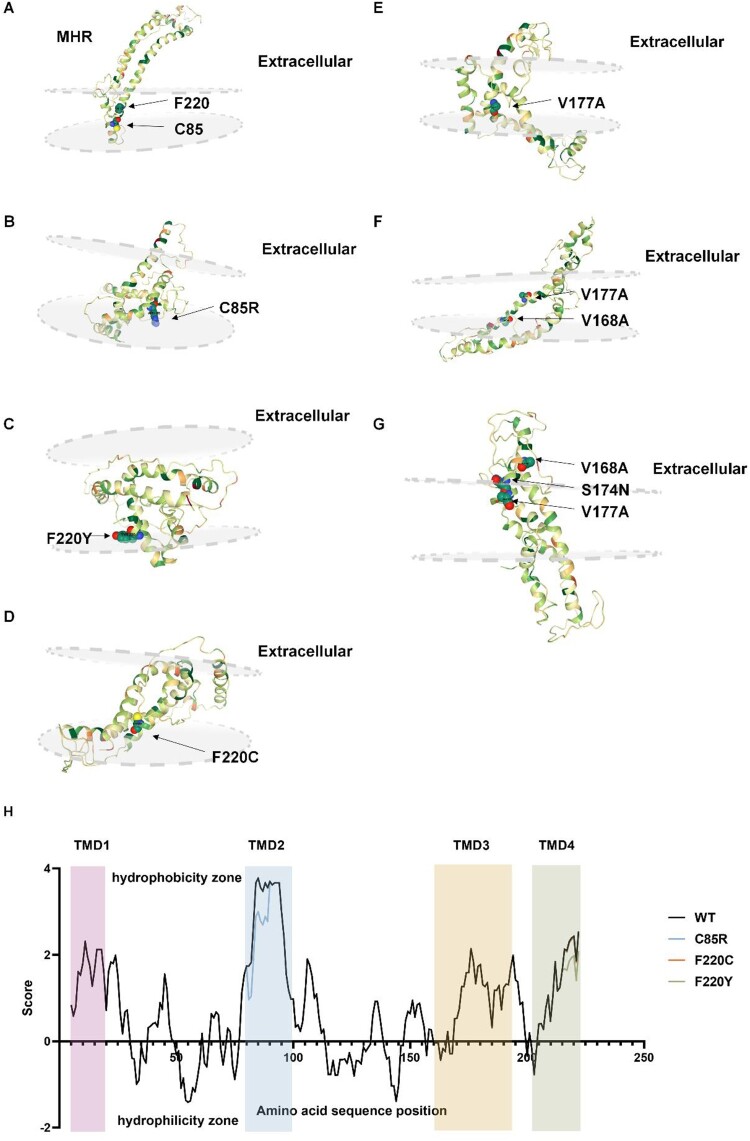
Structure predication of TMD mutated HBsAg. The focused sites are indicated by arrows and marked with locations and amino acid substitutions. **(A)** Structure prediction of wildtype S protein; **(B)** Structure prediction C85R mutated S protein; **(C and D)** Structure prediction of F220Y and F220C mutated S protein; **(E and G)** Structure prediction of S protein with TMD3 point mutation and combined mutations. **(H)** Analysis of hydrophilic and hydrophobic properties of wildtype and mutated HBsAg. The hydrophobicity score was determined for each HBsAg residue using the online software ExPASy ProtScale. Black line refers to the wildtype, blue line refers to the C85R, orange line to F220C and green line to F220Y. Four TMDs were marker with different background colour.

To explain the TMD mutations’ effect on the influence of HBsAg secretion, we used Phyre2 to predict the whole structure of S protein. Figure 6(A) supported that aa at position 85 and 220 located in the TMDs (between the phospholipid bilayer). When these two sites were replaced by hydrophilic aa (Figure 6B and D), the substituted aa would tend to penetrate out of the membrane. The whole structures became more clustered in TMDs, which supported the secretion disorder situation. However, structural prediction of three TMD3 mutant proteins (Figure 6E–G) showed no apparent secretion disturbance, suggesting the secretion and anchoring of S protein may be normal.

Expasy software was used to explore the hydrophilic and hydrophobic properties of the TMD region (Figure 6H). In the presence of C85R, the aa properties at this site changed from hydrophobic to hydrophilic, affecting the aa around this site and reducing the hydrophobicity of the entire TMD2. We speculated that the introduction of TMD mutation changed the properties as well as structure of S protein, leading to intracellular aggregation of HBsAg and secretion disorders.

### TMD mutations’ potential clinical significance

Finally, we explored the potential risks of OBI-related TMD mutations. Firstly, we explored the relationship between high-frequency TMD mutations and the serological pattern of carriers (Supplementary figure 6). In genotype B, 6 of the 22 selected mutations appeared more frequently in HBV surface antibody positive (anti-HBs+) OBI group (E164G, V168A + V177A, S174N + V177A, V168A + S174N + V177A, V168A + L175S + V177A, S174N + V177A + L175S). These mutated HBsAg may coexist with anti-HBs and cannot be recognized by antibodies, which is related to the selective pressure of antibodies. Among them, five mutations were combined mutations, which had greater structural changes and hindered the selection of antibodies.

Secondly, we explored the effects of mutated HBsAg on cell organelles. After detecting a series of endoplasmic reticulum (ER) stress-related proteins and proteins related to mitochondrial dysfunction. We detected the increase of mitochondrial fission regulatory protein DRP1 and ER stress-related protein ATF4 in cells expressing TMD mutant HBsAg. Results in supplementary figure 7 showed the DRP1 was increased when C85R mutated HBsAg aggregated inside the cells. F220C and F220Y mutant HBsAg both increased the expression of ATF4 and DRP1. The intracellular accumulation of TMD mutant HBsAg might trigger oxidative stress-related responses and affect the functions of endoplasmic reticulum and mitochondria.

## Discussion

Our study identified specific mutations in four transmembrane domains of HBsAg. Upon performing detailed functional analyses *in vitro* and *in vivo*, we revealed the potential mechanisms of these mutations involved in the development of OBI.

In genotype C, we selected nine high-frequency mutations and firstly reported that mutations V14G, R160S, E164G, V168A, P211L and C221R occurred with a higher frequency in OBI carriers. In genotype B, we first reported that I4T, A5E, G7R, G10R, K160R, E164A, E164G, S167L, F179Y, W182L, V184A, V184E, F220Y, and P217L were linked to genotype B OBI, which exhibited a higher occurrence rate in genotype B OBI patients. Based on the previous group, G10R [21], E164A [22], S167L [23], W182L [24], and F220Y [25] had been previously reported in genotype D OBI patients. Among them, E164A and W182L were discovered in OBI-related HCC patients. While in genotype A, F220C occurred in OBI strains from HCC patients [22]. Previous studies suggested that OBI-related mutations were connected with progressive liver diseases. With regard to this study, these mutations were discovered at high frequencies in genotype B, which may lead to the progression of OBI, and simultaneously pose a threat to OBI carriers.

Evidently, some of the OBI-related mutations identified in this study had been previously reported in the same genotype. In particular, V168A, S174N, L175S, V177A, and P178Q in genotype B occurred at high frequencies in the OBI group than in the HBsAg+ group [10,26,27]. S174N, L175S, and V177A [26,28] in genotype C existed with higher frequencies in the OBI group and were associated with undetected HBsAg. Consequently, our findings have supported the association of these mutations with OBI.

In genotype B, we found that the TMD mutations (C85R, F220C, and F220Y) showed impaired secretion capacity, which could be linked to OBI occurrence. We attempted to explain the specific mechanisms by which these mutations lead to OBI. In C85R, the original aa was replaced by the most hydrophilic arginine (R). This replacement would alter the hydrophilic properties of the 85^th^ site, thereby greatly affecting the structure and function of TMD2. At this specific site, arginine was prone to move from the hydrophobic phospholipid bilayer to the extracellular cytoplasm and retain the HBsAg, thereby affecting the HBsAg secretion. F220Y and F220C were substitutions at the same site. Three kinds of aa occurred at the 220th position, phenylalanine (F) is hydrophobic, cysteine amino acid (C) has yet to be classified, and tyrosine (Y) is a hydrophilic aa. Following the changes of hydrophobic properties of wildtype (F), the mutant HBsAg showed significant intracellular aggregation. We speculated that position 220 constitutes the key site associated with HBsAg anchoring. About this site, the introduction of various mutations comprising diverse properties can help determine its important role. Moreover, the site 220 anchors in the TMD4, which is a domain in doubt. Bioinformatics analysis hinted that C-terminal of S proteins contained two α helices and formed two transmembrane domains. Our findings further provided evidence for the existence and functions of both the C-terminal and TMD4.

The amino acid properties’ influence on HBV synthesis and expression were also discovered at other sites. For instance, A5E and A5T, E164G, and E164A, exhibited disparate expression levels with different amino acid replacement. In A5E and A5T, alanine (A) is replaced by glutamic acid (E) and threonine (T) respectively. Alanine is hydrophobic, while both glutamic acid and threonine are hydrophilic, with glutamic acid exhibiting higher hydrophilicity. Notably, the lower expression of A5E could correlate to greater changes in amino acid properties. Regarding E164G and E164A, the wild-type glutamic acid is hydrophilic, while the mutant alanine (A) and glycine (G) are hydrophobic, with alanine displaying greater hydrophobicity. Therefore, the lower expression of E164A might be also related to greater changes in amino acid properties. We speculated that mutation with greater difference in hydrophilic and hydrophobic properties exhibited lower HBsAg and HBV DNA levels. Furthermore, it was deduced that both pairs of mutations possessed lower HBsAg and HBV DNA when amino acids A and E were replaced with each other.

Combined mutations were assessed in this research. We discovered that V168A + V177A was highly expressed HBsAg and HBV DNA at extracellular level, which was significantly higher than the results of V177A and the V168A + S174N + V177A. Accordingly, V168A + V177A may have a higher RNA stability following the mutation, which would lead to the production and secretion of increased HBsAg. On the other hand, the prediction of protein structure may account for this uncommon serological pattern. Amongst these three mutations, V168A + V177A has the most unstable natural conformation, and could easily transit into V177A or V168A + S174N + V177A. In the OBI occurrence, all three mutations might display a state of coexistence, which could mitigate the extracellular HBsAg in the presence of V168A + V177A, and increase intracellular HBsAg in the presence of V177A or V168A + S174N + V177A, thereby approaching the classic OBI pattern of impaired secretion. We believed that the mutation combinations were more complicated in real OBI situations.

There were certain deficiencies in this study. First, the study was limited to samples collected in a cross-sectional study and restrained to specific regions of the HBV genome. In particular, most of our efforts emphasized combined mutations from only TMD3, while ignoring the interactions between TMDs. Ideally, longer cohort studies should be established to monitor the mutations during OBI development. Mutations in different gene regions may have an influence on HBsAg synthesis, secretion, and viral replication, which would require further evaluation by sequencing full length of HBV genome.

Second, high-frequency mutations from genotype C were not further explored in this study due to multiple mechanisms involved in the OBI occurrence. In genotype B, there were certain mutations which showed typical or special secretion patterns and required further evaluation. For instance, G10R and S174N + P178Q showed typical HBsAg secretion disorder. A5T, G7R, E164G, V168A, S174N, and L175S showed no significant decrease or even increase in extracellular HBsAg, but exhibited accumulation of intracellular HBsAg. However, we didn’t conduct a thorough assessment on the mechanisms of these mutations. Further studies will focus on the relationship of these mutations with OBI.

Third, we didn’t explore the interactions between different kinds of HBsAg: Small HBsAg protein (SHB), Middle HBsAg protein (MHB), and Large HBsAg protein (LHB). We found F220C and F220Y mutations expressed high level of LHB while their SHB was invisible. As we used plasmids contained HBV whole genome, we could not exclude the possibility that LHB may influence the expression of SHB. In our further studies, we are going to use both constructs containing HBV whole genome and S gene, the expression of S proteins and three kinds of HBsAg can support each other and interpret the experimental results in a better way.

For the *in vivo* study, we constructed animal models of HBV infection and serum HBsAg can be detected within 2 weeks. However, the time period was inadequate, we were unable to detect anti-HBs and explore the influence of mutated proteins on the immunity of mice. Constructing adenovirus-associated vectors containing HBV genome and establishing mouse models of chronic HBV infection can monitor serum HBsAg within 12 weeks, which would help identify real infection status of OBI.

Finally, we analysed the serological patterns related to TMD mutations, and preliminarily explored the association between TMD mutations and progressive liver disease, providing directions for further research. Primarily, TMD mutant HBsAg in genotype B was likely to coexist with anti-HBs and put pressure on antibody selection, thus challenges the likelihood of antibodies produced by vaccines potentially neutralizing these mutated HBsAg and the likely threat of the mutated HBV strains to the vaccinated population. Additionally, the TMD mutations increased the expression of mitochondrial fission regulatory protein and ER stress-related protein, indicating that these HBsAg affected normal functions of mitochondria and endoplasmic reticulum. By detecting the structure and function of mitochondria, exploring the downstream signaling pathway that HBsAg activated ER stress, we can lay foundations for the study of how OBI develops into progressive liver diseases.

In conclusion, this study collected real OBI samples, emphasized the TMDs relation to virus secretion, and constructed high-frequency TMD mutations based on plasmids containing whole HBV genomes. Cell and animal models were applied to detect HBsAg secretion. This study revealed the underlying mechanism involved in OBI occurrence and development, establishing a basis for clinical monitoring and treatment of OBI as well as OBI-related progressive liver diseases.

## Supplementary Material

Supplemental Material
