# Supplementary material for: Role of S protein transmembrane domain mutations in the development of occult hepatitis B virus infection
Source: Emerg Microbes Infect. 2022 Sep 21;11(1):2184–96. doi: 10.1080/22221751.2022.2114849 (PMC9518280; doi:10.1080/22221751.2022.2114849)
Supplement: Supplemental Material [file TEMI_A_2114849_SM1395.docx]

Role of S Protein Transmembrane Domain Mutations in the Development of Occult Hepatitis B Virus Infection

**Supporting Materials**

Supplementary Materials and Methods

Supplementary Figures and Legends

Supplementary Tables

Supplementary Materials and Methods

***Transfection and HBsAg quantitation***

Huh-7 cells in 6-well culture plate were maintained in DMEM with 10% fetal bovine serum and transfected with 2.5 μg HBV expressing plasmids we constructed when the density reached 90% with Lipofectamine 3000 (Invitrogen, USA). 72 hours later, supernatants were collected and cells were stored at -80°C. Extracellular HBsAg was detected using Abbott chemiluminescent microparticle immunoassays on the Abbott i2000 system (Abbott Diagnostic, USA). Plasmid pSELECT-zeo-SEAP (InvivoGen, USA) was co-transfected (500 ng/well) for normalization. Extracellular SEAP production was measured by QUANTI-Blue Solution kit (InvivoGen, USA). For all the mutants, at least 3 independent transfection experiments were performed. During transfection, mutant G145R is used as the positive control because its mutant proteins have impaired secretion and antibody binding[1,2]. I226S in genotype B is used as the negative control, showing a comparative secretion level compared to wildtype[3].

***Western blot analysis***

***For extracellular HBsAg analysis:*** Before ultracentrifugation, 2 mL 30% sucrose TNE solution (Bio-rad, GL1203, USA) were added to the bottom of the centrifuge tube. 5 mL cell culture supernatant were collected and added into the tube slowly, taking care not to disturb the layer of the TNE solution. After ultracentrifugation (50,000 rpm for 4 hours at 4°C with Thermo T-1270 rotor), culture supernatant and sucrose TNE solution were discarded. The translucent precipitation at the bottom of the centrifuge tube was resuspended with Laemmli loading buffer (Bio-rad, GL1372, USA) and boiled for western blot analysis using polyclonal rabbit anti-HBs antibody (Novus, NB100-62652, USA).

***For intracellular HBsAg analysis:*** Following transfection with HBV-expressing plasmids in Huh7 cells, cell pellet was lysed on ice for 30 minutes by NP-40 lysis buffer (Beyotime, P0013F, China) with 1/100 volume of protease inhibitors (Abcam, ab141032, USA). The cytosolic extracts were cleared by centrifugation at 12,000 rcf for 10 minutes at 4°C, and subjected to immunoblotting using primary polyclonal rabbit anti-HBs antibody (Novus, NB100-62652, USA), anti-beta Tubulin (Abcam, ab6046, USA) served as loading control. Goat Anti-Rabbit IgG H&L (HRP) (Abcam, ab205718, USA) was used as secondary antibody.

***For analysis of ER stress markers and Drp-1:*** Lysates were prepared on 72 hours post-transfection and subjected to immunoblotting as described above. Primary antibodies used were: ATF4 (Abcam, ab184909, USA) and Drp1 Ser616 (Abcam, ab184247, USA).

***Immunofluorescence assay***

The cells were inoculated into a 6-well plate with a concentration of 10^5^ cells/well. 48 hours after transfection, the plates were washed three times with PBS and fixed by 4% paraformaldehyde for 20 minutes, followed by permeabilization for 10 minutes at room-temperature with 0.25% Triton X-100. After incubation for 1 hour with 1% BSA for blockade of nonspecific binding, primary antibodies for HBsAg (FITC Anti-Hepatitis B Virus Surface Antigen antibody, Abcam, ab21021, USA) and endoplasmic reticulum (Alexa Fluor® 647 Anti-Calnexin antibody, Abcam, ab225062, USA) were added for 1h incubation at room-temperature. The plates were washed three times with PBS before 20-minute DAPI staining. Images were acquired using an OLYMPUS FV1000 confocal laser scanning biological microscope.

***Quantitative reverse transcription PCR***

Total RNA was extracted using QIAamp Viral RNA Mini Kit (QIAGEN, 52904, Germany) according to the manufacturer’s protocol. Real-time qRT-PCR was performed using Quantitative Detection Kit for Hepatitis B Virus Nucleic Acid (Livzon, PCR-Fluorescent Probe, China).

***In vivo analysis***

10 μg of HBV plasmid DNA were injected into the tail veins of C57BL/6 mice (male, 6 weeks old) within 8 s in a volume of PBS equivalent to 8% of the mouse body weight. Blood samples were collected from tail veins on day 1, 3, 5, 7 and 10 after injection. The HBsAg levels in the mouse serum from mutant or wildtype groups were detected using ELISA kit (Murex HBsAg Version 3, DiaSorin, Italy) according to the manufacturer’s protocol. On Day 10 after injection, the mice were killed for liver tissues to prepare frozen sections. Immunofluorescence assay for intracellular HBsAg was performed on the frozen section with primary antibodies for HBsAg (Abcam, ab21021, USA).

***Prediction software***

***PEP-FOLD software*** was used for the prediction of transmembrane domains (https://mobyle.rpbs.univ-paris-diderot.fr/cgi-bin/portal.py#forms::PEPFOLD3). PEP-FOLD can predict peptide structure from 5-50 amino acid sequences based on the coarse-grained model, and can list the most-native and lowest-energy conformations of the proteins.

***Phyre2 online analysis software*** can predict the tertiary structure of TMD mutant proteins (<http://www.sbg.bio.ic.ac.uk/phyre2>). After inputting the amino acid sequence to be predicted, intensive analysis can be selected. MolSTAR protein structure drawing software (https://www.novopro.cn/tools/molstar.html) can be used to optimize the results.

***Expasy online analysis software*** (http://www.expasy.org) was used to analyze the hydrophilic and hydrophobic properties of TMD mutants with secretion disorders. By selecting ***ProtScale analysis software***, inputting the amino acid sequence and name of the proteins, and choosing hydrophilic and hydrophobic property analysis, the hydrophobic score of each site and hydrophobic property of the whole sequence can be obtained.

***Statistical analysis***

SPSS 21.0 software and GraphPad Prism 8.0 were utilized for statistical analysis. Fisher's exact test was applied to compare the OBI-related high-frequency mutations between OBI and HBsAg+ group. Kruskal-Wallis analysis was used for the comparisons of differences among TMD mutants and wildtype on extracellular and intracellular HBsAg. p<0.05 (two-tailed) was considered statistically significant. Fisher's exact test and co-variation analysis were performed on paired mutations from monoclonal sequences. Coefficient of binomial correlation (Phi) was calculated to evaluate the correlation. Co-variations with 0.3<Phi<1 and p<0.05 were considered as statistically significant positive correlated mutations.

***Ethical approval***

This study was conformed to the ethical guidelines of the 1975 Declaration of Helsinki and was reviewed and approved by the Medical Ethical Committee of Beijing Hospital (2017BJYYEC-138-01). Written informed consent was obtained from each enrolled donor before donation.

Supplementary Figures and Legends


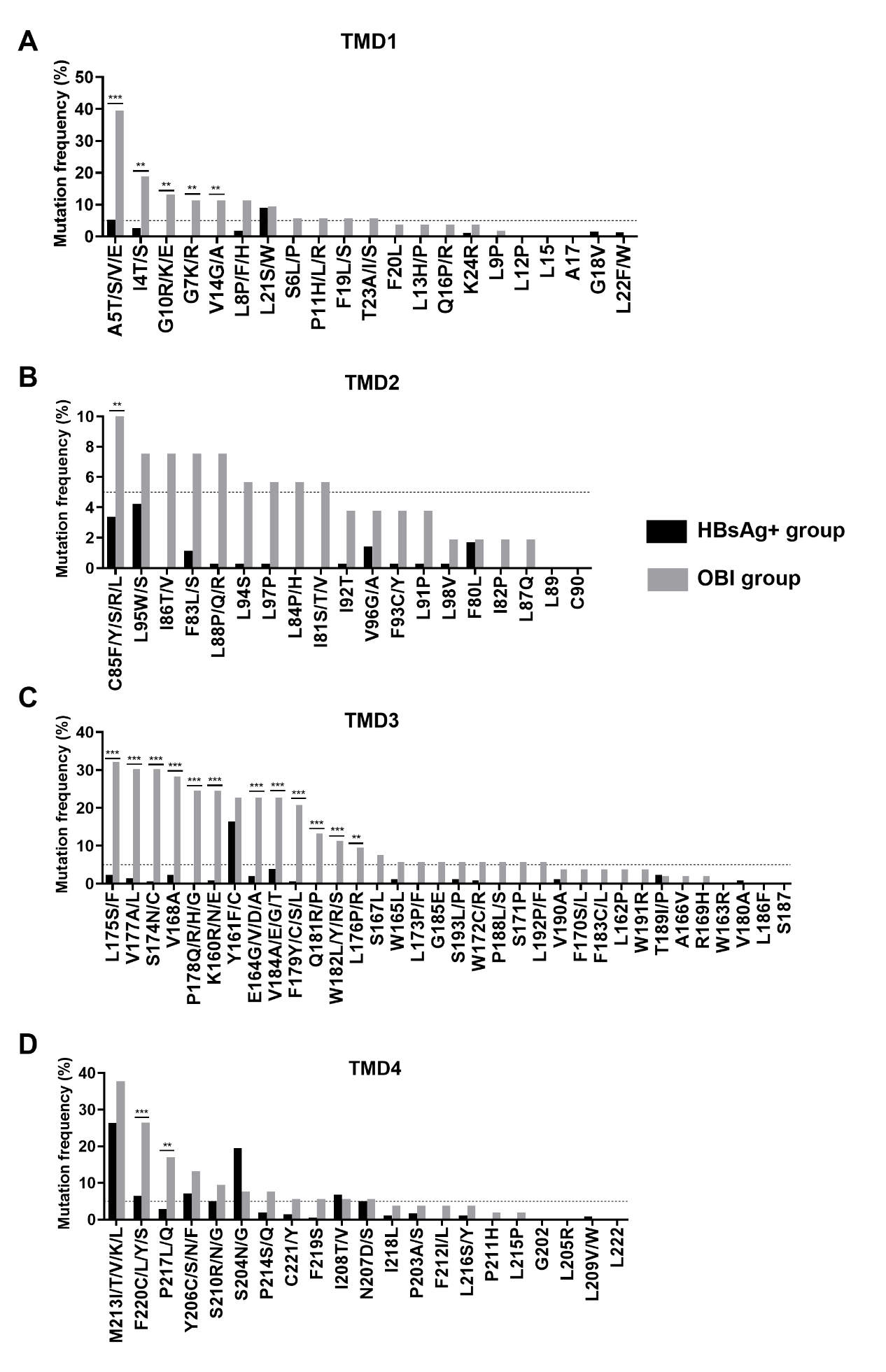


**Supplementary figure 1. Selection for high-frequency mutations in four TMDs of genotype B OBI sequences.** OBI-related mutations between OBI and HBsAg+ control group were examined using Fisher's exact test (two-sided). Mutations that were statistically appeared with high frequency in OBI group were included in subsequent studies. The positions of four TMDs were showing below: TMD1 (4-24 aa), TMD2 (80-98 aa), TMD3 (160-193 aa) and TMD4 (202-222aa). Each site of TMD may be substituted with various amino acids. * p < 0.05, ** p < 0.01, *** p < 0.001. Abbreviation: TMD, Transmembrane domain; OBI, Occult hepatitis B virus infection.


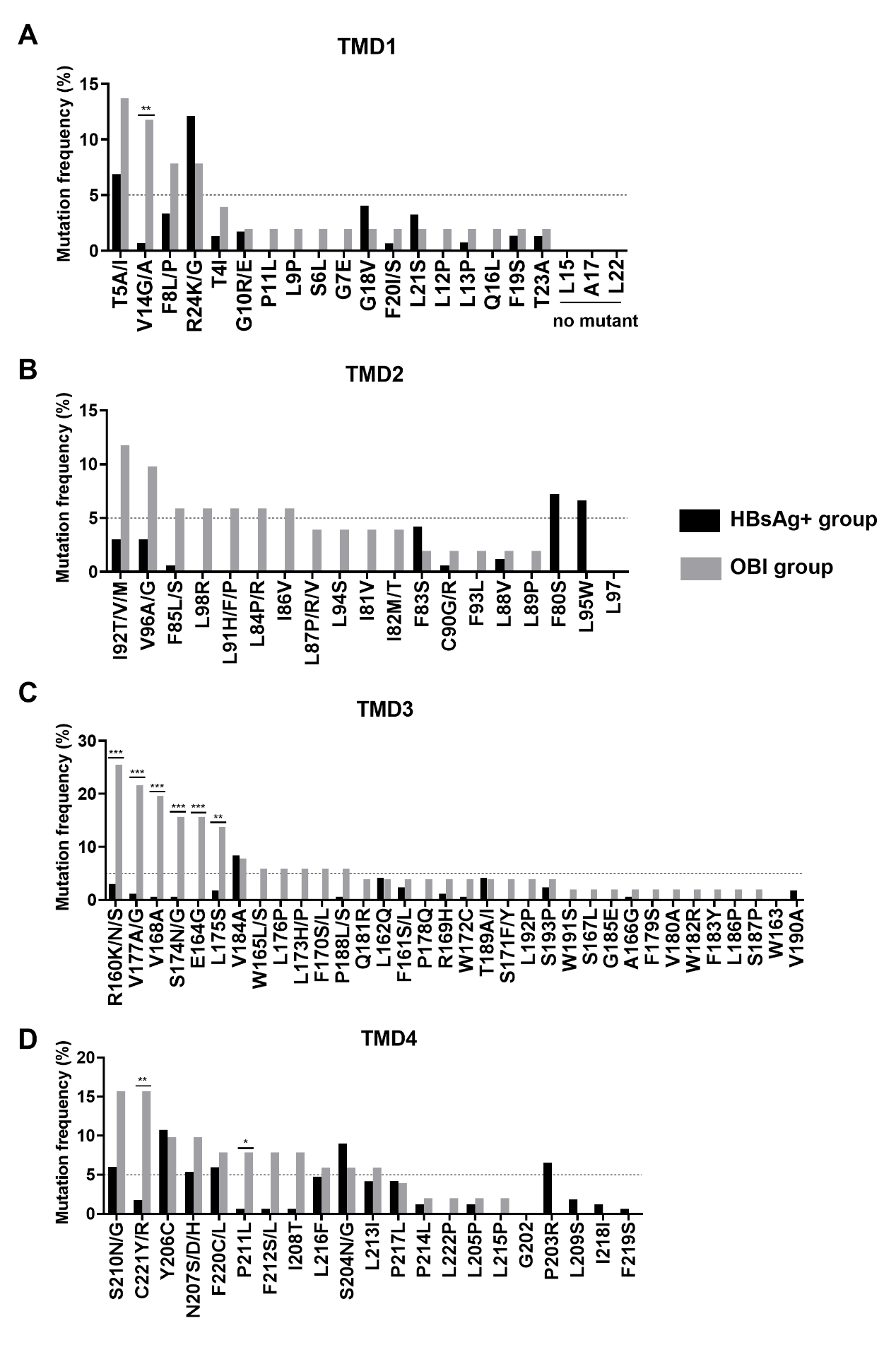


**Supplementary figure 2. Selection for high-frequency mutations in four TMDs of genotype C OBI sequences.** OBI-related mutations between OBI and HBsAg+ control group were examined using Fisher's exact test (two-sided). Mutations that were statistically appeared with high frequency in OBI group were included in subsequent studies. The positions of four TMDs were showing below: TMD1 (4-24 aa), TMD2 (80-98 aa), TMD3 (160-193 aa) and TMD4 (202-222aa). Each site of TMD may be substituted with various amino acids. * p < 0.05, ** p < 0.01, *** p < 0.001. Abbreviation: TMD, Transmembrane domain; OBI, Occult hepatitis B virus infection.

**Supplementary figure 3. High-frequency mutations in four TMDs of genotype C OBI sequences.** OBI-related mutations between OBI and HBsAg+ control group were examined using Fisher's exact test (two-sided). Mutations from four TMDs that were statistically appeared with high-frequency in OBI group were included in subsequent studies, including 1 site in TMD1 (the 14^th^ site), 6 sites in TMD3 (the 160^th^, 164^th^, 168^th^, 174^th^, 175^th^ and 177^th^ site), and 2 sites in TMD4 (the 211^th^ and 221^th^ site). Each site of these TMDs may be substituted with various amino acids. The positions of four TMDs: TMD1 (4-24 aa), TMD2 (80-98 aa), TMD3 (160-193 aa) and TMD4 (202-222aa). Abbreviation: TMD, Transmembrane domain; OBI, Occult hepatitis B virus infection.


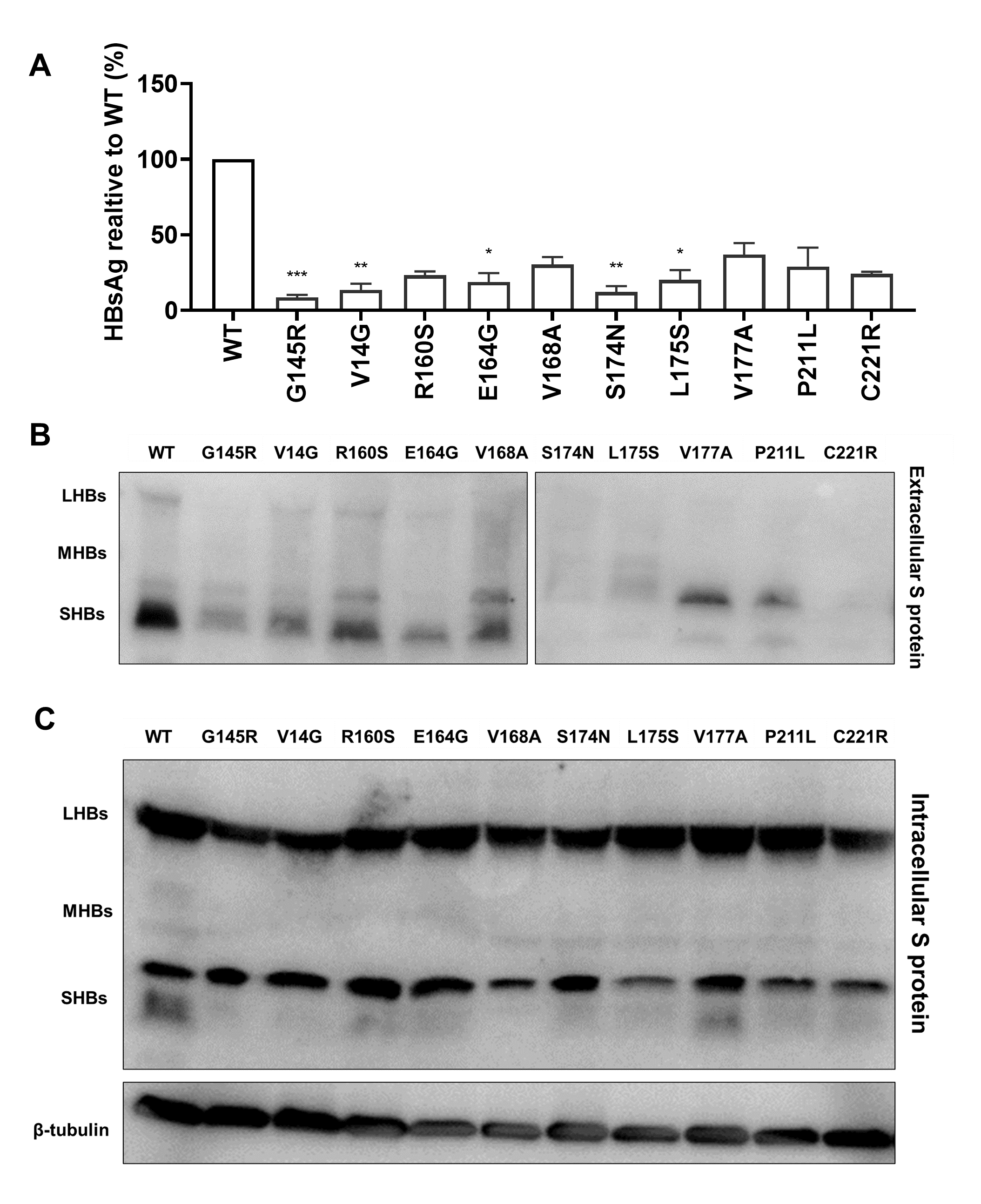


**Supplementary figure 4. Functional analysis of TMD mutations in genotype C.** **(A)** Extracellular HBsAg expression of wildtype or TMD mutants was detected by chemiluminescent immunoassay (CLIA) and normalized according to SEAP activity. Data were demonstrated as means (SD) from three independent experiments and examined by Kruskal-Wallis analysis. * p < 0.05, ** p < 0.01, *** p < 0.001. Western blot (WB) analysis was performed to detect extracellular **(B)** and intracellular **(C)** HBsAg of wildtype and TMD mutations in genotype C. Abbreviation: WT, Wildtype; HBsAg, Hepatitis B virus antigen; LHB, Large HBsAg; MHB, Middle HBsAg; SHB, Small HBsAg.

**Supplementary figure 5. The level of HBV DNA in culture supernatant detected by qPCR (Genotype C)**. Extracellular HBV DNA expressions of 11 samples of genotype C (9 TMD mutations, positive control G145R and wildtype) were shown. The HBV DNA level was calculated with three independent experiments.


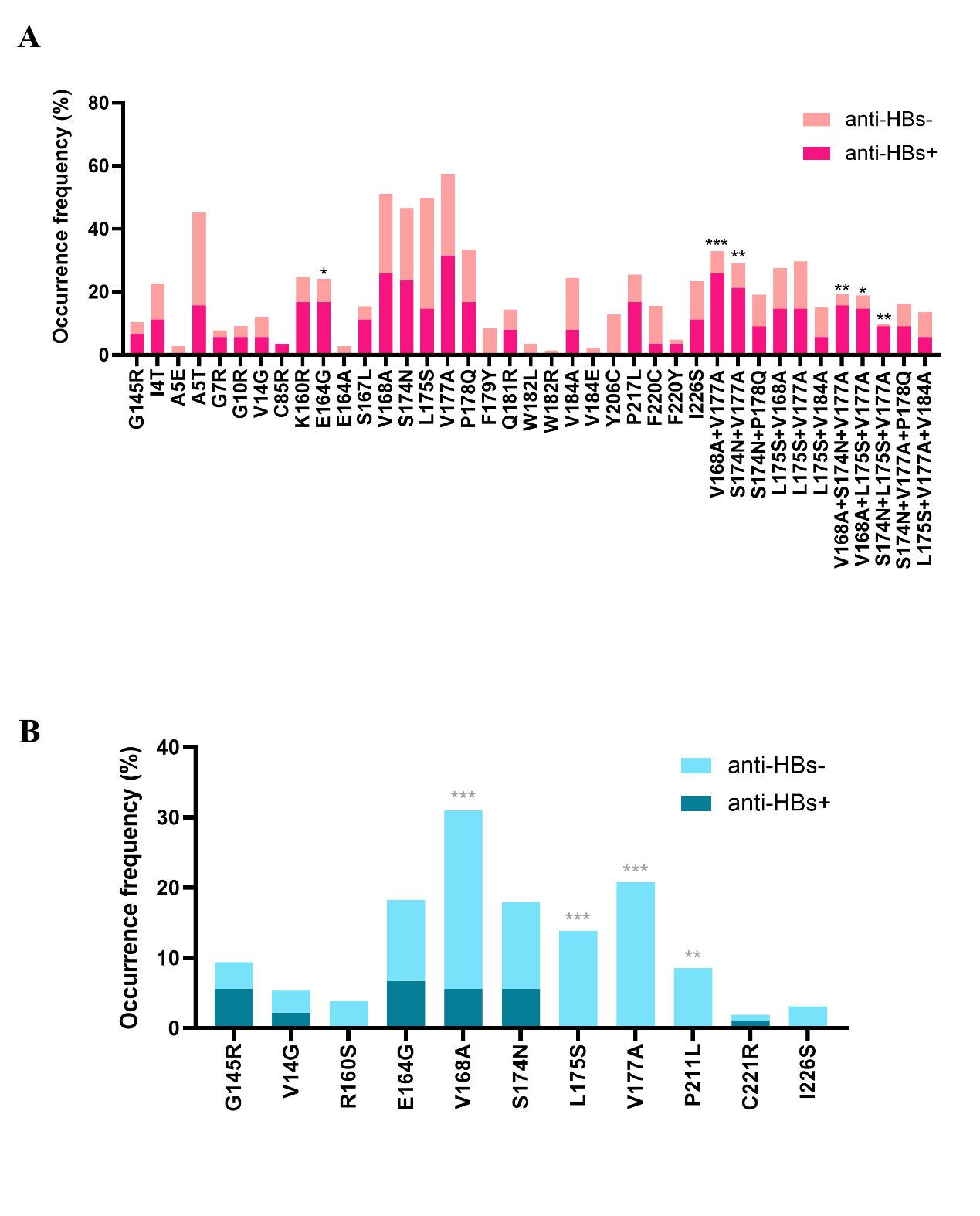


**Supplementary figure 6. Relationship between transmembrane domain mutations and anti-HBs in serum.** This study selected high-frequency TMD mutations from genotype B **(A)** and genotype C **(B)**, calculated the occurrence rates of these TMD mutations in anti-HBs positive and anti-HBs negative samples with Pearson Chi-Square test. * p < 0.05, ** p < 0.01, *** p < 0.001. Abbreviation: anti-HBs, Hepatitis B virus surface antibody.

**
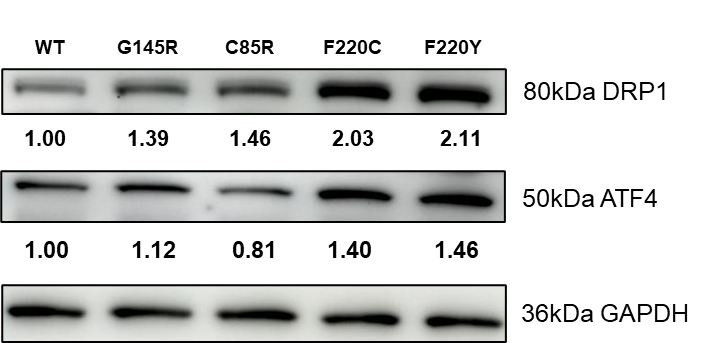
**

**Supplementary figure 7. Expression of intracellular organelle-related proteins after transfection with TMD mutant plasmids.** Western blot (WB) analysis was performed to detect intracellular DRP1 and ATF4 expression after transfection with wildtype, positive control, or TMD mutant plasmids in Huh-7 cells. After normalizing with reference gene GAPDH, the number below each stripe indicates their density compared to wildtype (The density of wildtype is 1.00.). Abbreviation: DRP1, Dynamin-related protein 1; ATF4, Activating transcription factor 4.


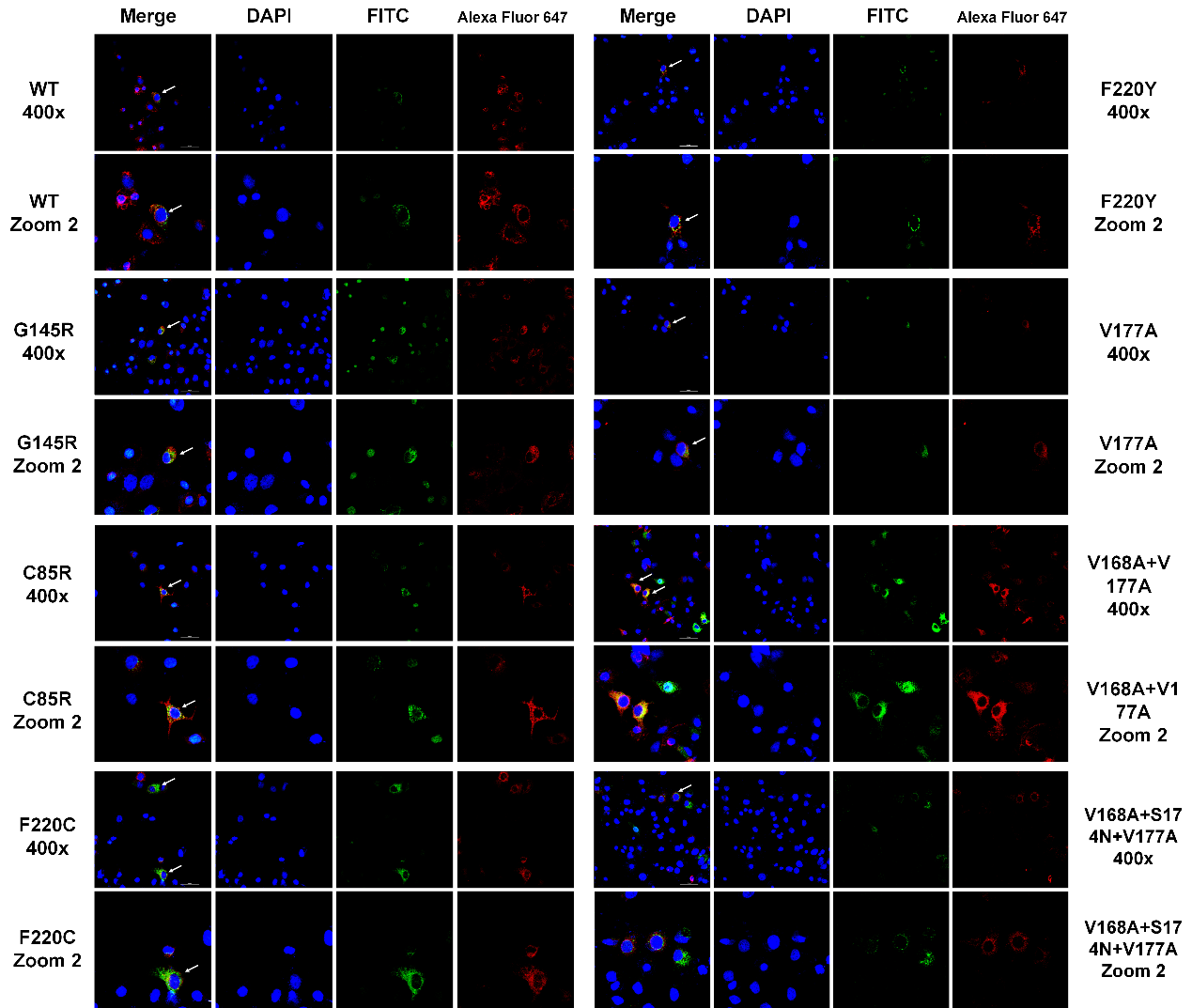


**Supplementary figure 8. Detection of intracellular HBsAg with immunofluorescence assays.** Results of dual fluorescence immunostaining for intracellular HBsAg (FITC, green) and endoplasmic reticulum calnexin marker (Alexa Fluor 647, red) were shown. The nucleus of cells was stained with DAPI. The scale bar stands for 50 μm. Based on the ×400 magnification, the visual field was zoom in 2 times (Zoom 2) to get a closer observation of cells.

**Supplementary figure 9. ELISA (A) and CLIA (B) detection for HBsAg in culture supernatant (Genotype C)**. Data were demonstrated as means (SD) from three independent experiments and examined by Kruskal-Wallis analysis. * p < 0.05, ** p < 0.01, *** p < 0.001.

**Supplementary figure 10. WB band density analysis for HBsAg in cell lysate (Genotype C).** Data were demonstrated as means (SD) from three independent experiments.


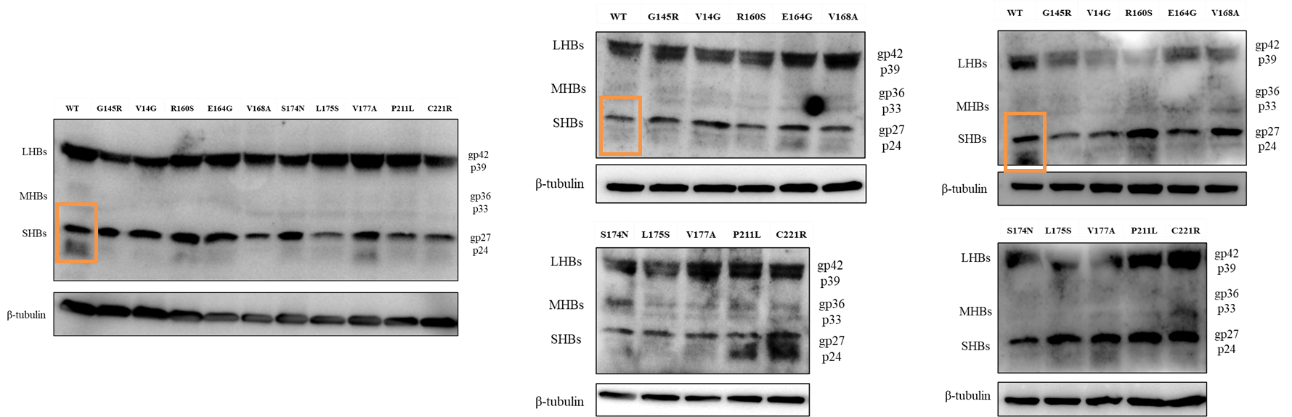


**Supplementary figure 11. Three independent WB analysis for HBsAg in cell lysate** (Genotype C, Orange box indicate wildtype S proteins)


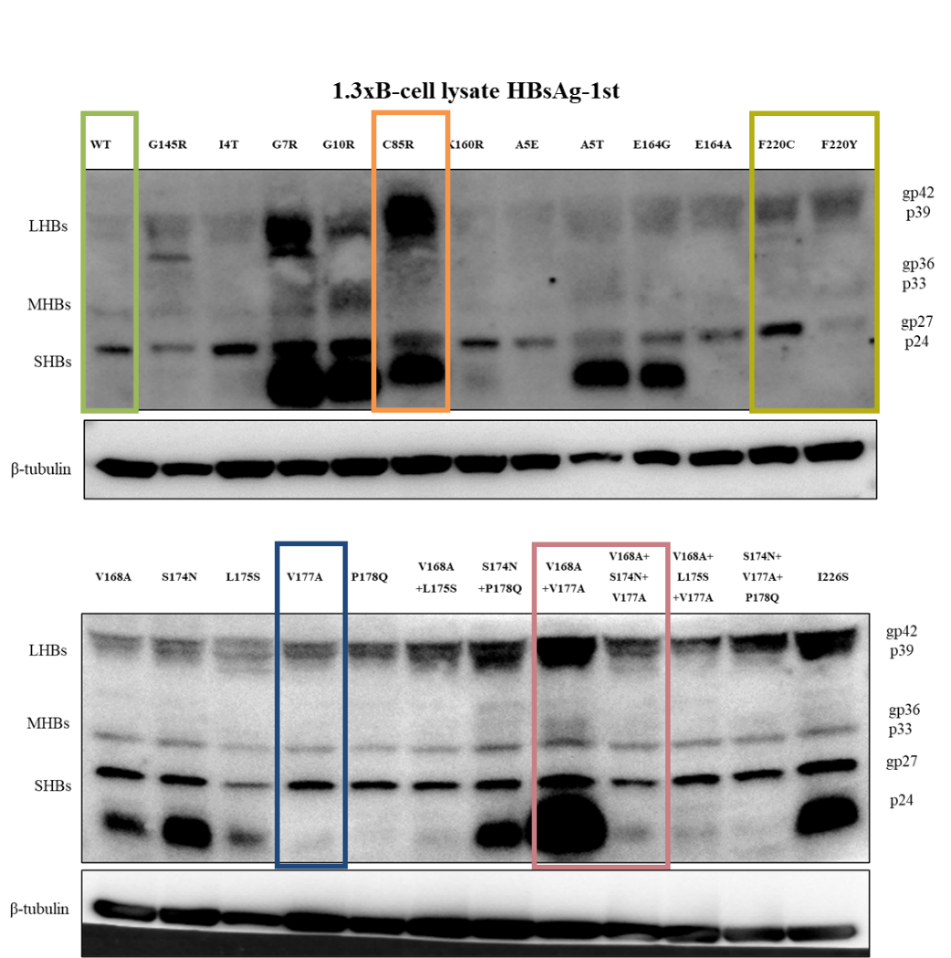


**Supplementary figure 12-1. Three independent WB analysis for HBsAg in cell lysate** (Genotype B, boxes with different color indicate specific mutations we selected, first time)


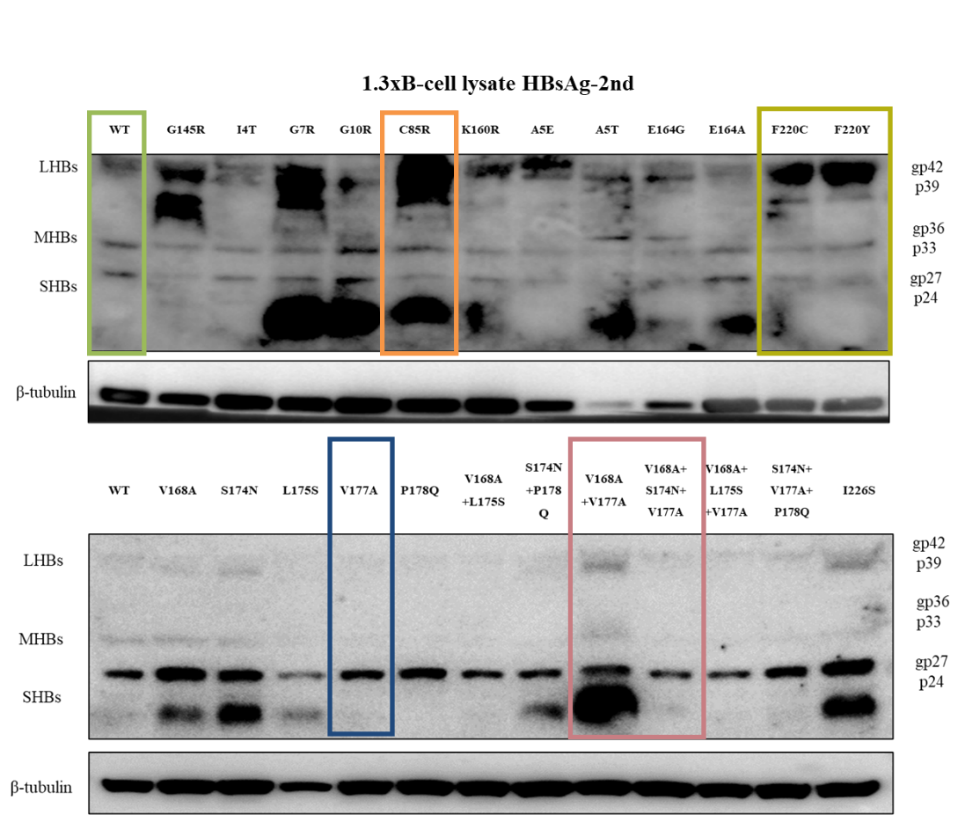


**Supplementary figure 12-2. Three independent WB analysis for HBsAg in cell lysate** (Genotype B, boxes with different color indicate specific mutations we selected, second time)


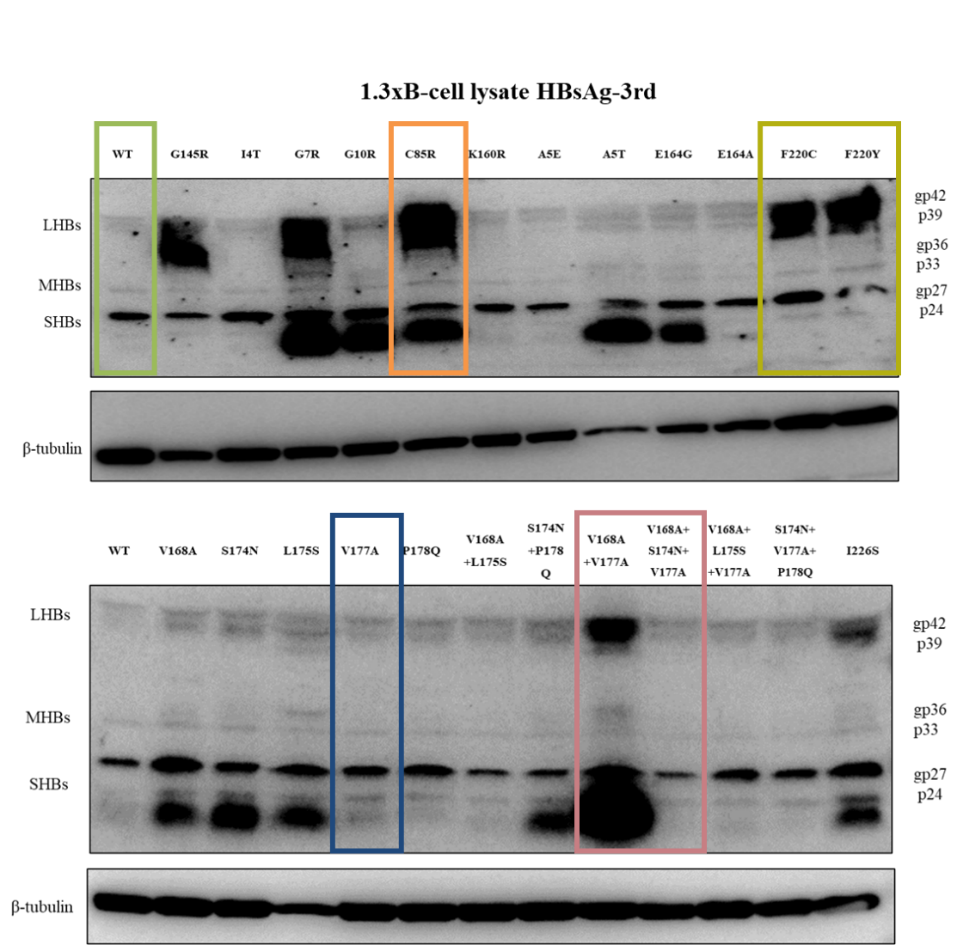


**Supplementary figure 12-3. Three independent WB analysis for HBsAg in cell lysate** (Genotype B, boxes with different color indicate specific mutations we selected, third time)

**Supplementary figure 13. Band density of mutant and wildtype HBsAg in cell lysate (Genotype B).** Data were demonstrated as means (SD) from three independent experiments and examined by Kruskal-Wallis analysis. * p < 0.05, ** p < 0.01, *** p < 0.001.


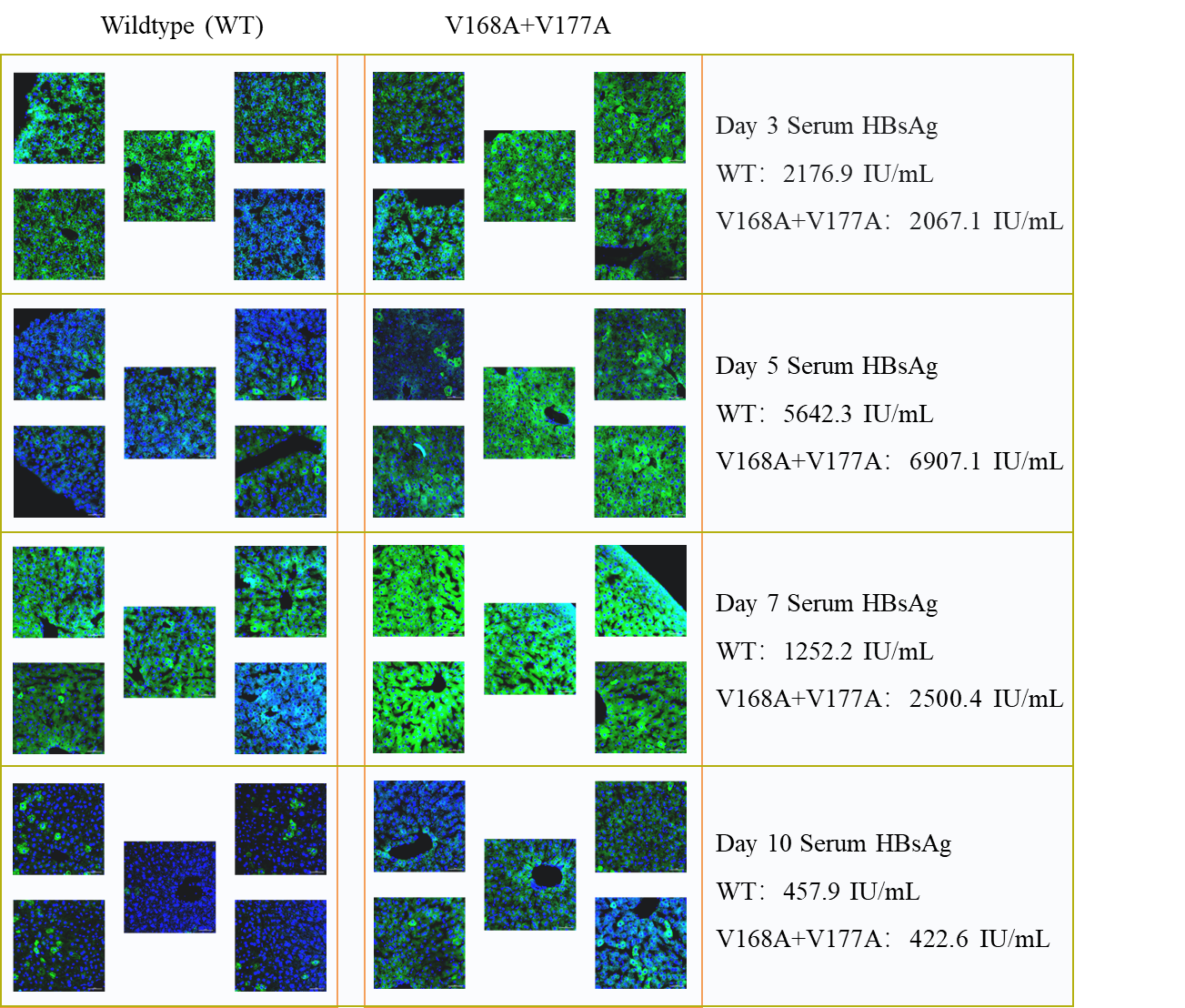


**Supplementary figure 14. Comparison of HBsAg in liver tissues between wildtype and V168A+V177A.** Six C57BL/6 mice were included in each group to construct HBV infection model by hydrodynamic injection with wildtype or V168A+V177A mutant plasmids. Mice in each group were sacrificed at specific time point for liver tissue. Frozen sections were stained with immunofluorescence assay, HBsAg was stained with FITC (green), and nuclei were stained with DAPI (blue). Confocal microscopy was used to capture the pictures (magnification ×400), and **we chose five horizons from each mouse**. Serum HBsAg in the mice from specific group was labeled next to the pictures.

Supplementary Tables

Supplementary table 1. Site-directed mutagenesis primers for genotype C HBV

| Primer name | Primer sequences |
| --- | --- |
| HBV S gene sequencing | TTTCCTGCTGGTGGCTCCAGTT |
| 1.2xC-G145R-F | GCTGTACAAAACCTTCGGACAGAAACTGCACTTGTATTCCCATC |
| 1.2xC-G145R-R | GAATACAAGTGCAGTTTCTGTCCGAAGGTTTTGTACAGCAAC |
| 1.2xC-V14G-F | TCCTAGGACCCCTGCTCGGGTTACAGGCGGGGTTTTTCTTGT |
| 1.2xC-V14G-R | GAAAAACCCCGCCTGTAACCCGAGCAGGGGTCCTAGGAATCC |
| 1.2xC-R160S-F | ATCCTGGGCTTTCGCAAGCTTCCTATGGGAGTGGGCCTCAGT |
| 1.2xC-R160S-R | CCCACTCCCATAGGAAGCTTGCGAAAGCCCAGGATGATGG |
| 1.2xC-E164G-F | TCGCAAGATTCCTATGGGGGTGGGCCTCAGTCCGTTTCTCCT |
| 1.2xC-E164G-R | AAACGGACTGAGGCCCACCCCCATAGGAATCTTGCGAAAGCC |
| 1.2xC-V168A-F | TATGGGAGTGGGCCTCAGCCCGTTTCTCCTGGCTCAGTTTAC |
| 1.2xC-V168A-R | CTGAGCCAGGAGAAACGGGCTGAGGCCCACTCCCATAGGAAT |
| 1.2xC-S174N-F | TCCGTTTCTCCTGGCTCAATTTACTAGTGCCATTTGTTCAGT |
| 1.2xC-S174N-R | ACAAATGGCACTAGTAAATTGAGCCAGGAGAAACGGACTGAG |
| 1.2xC-L175S-F | CGTTTCTCCTGGCTCAGTTCACTAGTGCCATTTGTTCAGTGGT |
| 1.2xC-L175S-R | TGAACAAATGGCACTAGTGAACTGAGCCAGGAGAAACGGACT |
| 1.2xC-V177G-F | CCTGGCTCAGTTTACTAGGGCCATTTGTTCAGTGGTTCGTAG |
| 1.2xC-V177G-R | AACCACTGAACAAATGGCCCTAGTAAACTGAGCCAGGAGAAA |
| 1.2xC-P211L-F | TGTACAACATCTTGAGTCTCTTTTTACCTCTATTACCAATTT |
| 1.2xC-P211L-R | GGTAATAGAGGTAAAAAGAGACTCAAGATGTTGTACAGACTT |
| 1.2xC-C221R-F | CTATTACCAATTTTCTTTCGTCTTTGGGTATACATTTGAACC |
| 1.2xC-C221R-R | AAATGTATACCCAAAGACGAAAGAAAATTGGTAATAGAGGTA |
| 1.2xC-I226S-F | TTTGTCTTTGGGTATACAGTTGAACCCCAATAAAACCAAACG |
| 1.2xC-I226S-R | GGTTTTATTGGGGTTCAACTGTATACCCAAAGACAAAAGAAA |

Note: the suffix F after the primer names stands for Forward primer, and suffix R stands for Reverse primer.

Supplementary table 2. Site-directed mutagenesis primers for genotype B HBV

| Primer name | Primer sequences |
| --- | --- |
| 1.3xB-A5E-F | CCGAACATGGAGAACATCGAATCAGGACTCCTAGGACCCCTG |
| 1.3xB-A5E-R | GGTCCTAGGAGTCCTGATTCGATGTTCTCCATGTTCGGTACA |
| 1.3xB-A5T-F | CCGAACATGGAGAACATCACATCAGGACTCCTAGGACCCCTG |
| 1.3xB-A5T-R | GTCCTAGGAGTCCTGATGTGATGTTCTCCATGTTCGGTACAG |
| 1.3xB-I4T-F | TACCGAACATGGAGAACACCGCATCAGGACTCCTAGGACCCC |
| 1.3xB-I4T-R | CCTAGGAGTCCTGATGCGGTGTTCTCCATGTTCGGTACAGGG |
| 1.3xB-G7R-F | ATGGAGAACATCGCATCAAGACTCCTAGGACCCCTGCTCGTG |
| 1.3xB-G7R-R | GCAGGGGTCCTAGGAGTCTTGATGCGATGTTCTCCATGTTCG |
| 1.3xB-V14G-F | TCCTAGGACCCCTGCTCGGGTTACAGGCGGGGTTTTTCTTGT |
| 1.3xB-V14G-R | AAAAACCCCGCCTGTAACCCGAGCAGGGGTCCTAGGAGTCCT |
| 1.3xB-G10R-F | ATCGCATCAGGACTCCTAAGACCCCTGCTCGTGTTACAGGCG |
| 1.3xB-G10R-R | GTAACACGAGCAGGGGTCTTAGGAGTCCTGATGCGATGTTCT |
| 1.3xB-C85R-F | CGTTTTATCATCTTCCTCCGCATCCTGCTGCTATGCCTCATC |
| 1.3xB-C85R-R | GGCATAGCAGCAGGATGCGGAGGAAGATGATAAAACGCCGCA |
| 1.3xB-V177A-F | CTTGGCTCAGTTTACTAGCGCAATTTGTTCAGTGGTTCGTAG |
| 1.3xB-V177A-R | AACCACTGAACAAACGGCGCTAGTAAACTGAGCCAAGAGAAA |
| 1.3xB-L175S-F | GTTTCTCTTGGCTCAGTTCACTAGTGCCGTTTGTTCAGTGGT |
| 1.3xB-L175S-R | TGAACAAACGGCACTAGTGAACTGAGCCAAGAGAAACGGACT |
| 1.3xB-S174N-F | GTCCGTTTCTCTTGGCTCAATTTACTAGTGCCGTTTGTTCAG |
| 1.3xB-S174N-R | CAAACGGCACTAGTAAATTGAGCCAAGAGAAACGGACTGAGG |
| 1.3xB-P178Q-F | GGCTCAGTTTACTAGTGCAATTTGTTCAGTGGTTCGTAGGGC |
| 1.3xB-P178Q-R | TACGAACCACTGAACAAATTGCACTAGTAAACTGAGCCAAGA |
| 1.3xB-K160R-F | CATCTTGGGCTTTCGCAAGATACCTATGGGAGTGGGCCTCAG |
| 1.3xB-K160R-R | GCCCACTCCCATAGGTATCTTGCGAAAGCCCAAGATGATGGG |
| 1.3xB-E164G-F | TCGCAAAATACCTATGGGGGTGGGCCTCAGTCCGTTTCTCTT |
| 1.3xB-E164G-R | AAACGGACTGAGGCCCACCCCCATAGGTATTTTGCGAAAGCC |
| 1.3xB-E164A-F | TCGCAAAATACCTATGGGCGTGGGCCTCAGTCCGTTTCTCTT |
| 1.3xB-E164A-R | AAACGGACTGAGGCCCACGCCCATAGGTATTTTGCGAAAGCC |
| 1.3xB-V184A-F | CGTTTGTTCAGTGGTTCGCAGGGCTTTCCCCCACTGTCTGGC |
| 1.3xB-V184A-R | ACAGTGGGGGAAAGCCCTGCGAACCACTGAACAAACGGCACT |
| 1.3xB-V184E-F | CGTTTGTTCAGTGGTTCGAAGGGCTTTCCCCCACTGTCTGGC |
| 1.3xB-V184E-R | ACAGTGGGGGAAAGCCCTTCGAACCACTGAACAAACGGCACT |
| 1.3xB-F179Y-F | TCAGTTTACTAGTGCCGTATGTTCAGTGGTTCGTAGGGCTTT |
| 1.3xB-F179Y-R | CCTACGAACCACTGAACATACGGCACTAGTAAACTGAGCCAA |
| 1.3xB-Q181R-F | TACTAGTGCCGTTTGTTCGGTGGTTCGTAGGGCTTTCCCCCA |
| 1.3xB-Q181R-R | GAAAGCCCTACGAACCACCGAACAAACGGCACTAGTAAACTG |
| 1.3xB-S167L-F | ACCTATGGGAGTGGGCCTTAGTCCGTTTCTCTTGGCTCAGTT |
| 1.3xB-S167L-R | AGCCAAGAGAAACGGACTAAGGCCCACTCCCATAGGTATTTT |
| 1.3xB-W182L-F | CTAGTGCCGTTTGTTCAGTTGTTCGTAGGGCTTTCCCCCACT |
| 1.3xB-W182L-R | GGGAAAGCCCTACGAACAACTGAACAAACGGCACTAGTAAAC |
| 1.3xB-W182R-F | CTAGTGCCGTTTGTTCAGCGGTTCGTAGGGCTTTCCCCCACT |
| 1.3xB-W182R-R | GGGAAAGCCCTACGAACCGCTGAACAAACGGCACTAGTAAAC |
| 1.3xB-F220C-F | CGCTGTTACCAATTTTCTGTTGTCTTTGGGTATACATTTAAA |
| 1.3xB-F220C-R | ATGTATACCCAAAGACAACAGAAAATTGGTAACAGCGGCATA |
| 1.3xB-F220Y-F | CGCTGTTACCAATTTTCTATTGTCTTTGGGTATACATTTAAA |
| 1.3xB-F220Y-R | ATGTATACCCAAAGACAATAGAAAATTGGTAACAGCGGCATA |
| 1.3xB-Y206C-F | ATTGGGGGCCAAGTCTGTGCAACATCTTGAGTCCCTTTATGC |
| 1.3xB-Y206C-R | AAGGGACTCAAGATGTTGCACAGACTTGGCCCCCAATACCAC |
| 1.3xB-P217L-F | CCTTTATGCCGCTGTTACTAATTTTCTTTTGTCTTTGGGTAT |
| 1.3xB-P217L-R | CAAAGACAAAAGAAAATTAGTAACAGCGGCATAAAGGGACTC |
| 1.3xB-G145R-F | TGTACAAAACCTACGGACAGAAACTGCACCTGTATTCCCATC |
| 1.3xB-G145R-R | GAATACAGGTGCAGTTTCTGTCCGTAGGTTTTGTACAGCAAC |
| 1.3xB-I226S-F | TTTGTCTTTGGGTATACAGTTAAACCCTCACAAAACAAAAAG |
| 1.3xB-I226S-R | TGTTTTGTGAGGGTTTAACTGTATACCCAAAGACAAAAGAAA |

Note: the suffix F after the primer names stands for Forward primer, and suffix R stands for Reverse primer.

Supplementary table 3. Multiple site-directed mutagenesis primers for genotype B HBV

| Primer name | Primer sequences |
| --- | --- |
| S174N+V177A-F | CTTGGCTCAATTTACTAGCGCCGTTTGTTCAGTGGTTCGTAG |
| S174N+V177A-R | AACCACTGAACAAACGGCGCTAGTAAATTGAGCCAAGAGAAA |
| S174N+P178Q-F | GGCTCAATTTACTAGTGCAATTTGTTCAGTGGTTCGTAGGGC |
| S174N+P178Q-R | TACGAACCACTGAACAAATTGCACTAGTAAATTGAGCCAAGA |
| L175S+V177A-F | CTTGGCTCAGTTCACTAGCGCCGTTTGTTCAGTGGTTCGTAG |
| L175S+V177A-R | AACCACTGAACAAACGGCGCTAGTGAACTGAGCCAAGAGAAA |
| L175S+V184A-F | CGTTTGTTCAGTGGTTCGCAGGGCTTTCCCCCACTGTCTGGC |
| L175S+V184A-R | ACAGTGGGGGAAAGCCCTGCGAACCACTGAACAAACGGCACT |
| L175S+V168A-F | TATGGGAGTGGGCCTCAGCCCGTTTCTCTTGGCTCAGTTCAC |
| L175S+V168A-R | CTGAGCCAAGAGAAACGGGCTGAGGCCCACTCCCATAGGTAT |
| V168A+V177A-F | CTTGGCTCAGTTTACTAGCGCCGTTTGTTCAGTGGTTCGTAG |
| V168A+V177A-R | AACCACTGAACAAACGGCGCTAGTAAACTGAGCCAAGAGAAA |
| V168A+L175S+V177A-F | TATGGGAGTGGGCCTCAGCCCGTTTCTCTTGGCTCAGTTCAC |
| V168A+L175S+V177A-R | CTGAGCCAAGAGAAACGGGCTGAGGCCCACTCCCATAGGTAT |
| S174N+V177A+P178Q-F | CTTGGCTCAATTTACTAGCGCAATTTGTTCAGTGGTTCGTAG |
| S174N+V177A+P178Q-R | AACCACTGAACAAATTGCGCTAGTAAATTGAGCCAAGAGAAA |
| V168A+S174N+V177A-F | CTTGGCTCAATTTACTAGCGCCGTTTGTTCAGTGGTTCGTAG |
| V168A+S174N+V177A-R | AACCACTGAACAAACGGCGCTAGTAAATTGAGCCAAGAGAAA |
| L175S+V177A+V184A-F | CGTTTGTTCAGTGGTTCGCAGGGCTTTCCCCCACTGTCTGGC |
| L175S+V177A+V184A-R | ACAGTGGGGGAAAGCCCTGCGAACCACTGAACAAACGGCGCT |
| S174N+L175S+V177A-F | CTTGGCTCAATTCACTAGCGCCGTTTGTTCAGTGGTTCGTAG |
| S174N+L175S+V177A-R | AACCACTGAACAAACGGCGCTAGTGAATTGAGCCAAGAGAAA |

Note: the suffix F after the primer names stands for Forward primer, and suffix R stands for Reverse primer.

**References**

1. Wu C, Deng W, Deng L, Cao L, Qin B, Li S, et al. Amino Acid Substitutions at Positions 122 and 145 of Hepatitis B Virus Surface Antigen (HBsAg) Determine the Antigenicity and Immunogenicity of HBsAg and Influence In Vivo HBsAg Clearance. J Virol. 2012 Apr 15;86(8):4658–69.
2. Kalinina T. Deficiency in virion secretion and decreased stability of the hepatitis B virus immune escape mutant G145R. Hepatology. 2003 Nov;38(5):1274–81.
3. Wang H, Wang M, Huang J, Xu R, Liao Q, Shan Z, et al. Novel hepatitis B virus surface antigen mutations associated with occult genotype B hepatitis B virus infection affect HBsAg detection. J Viral Hepat. 2020 Sep;27(9):915–21.
